# Chl1 DNA Helicase Regulates Scc2 Deposition Specifically during DNA-Replication in *Saccharomyces cerevisiae*

**DOI:** 10.1371/journal.pone.0075435

**Published:** 2013-09-26

**Authors:** Soumya Rudra, Robert V. Skibbens

**Affiliations:** Department of Biological Sciences, Lehigh University, Bethlehem, Pennsylvania, United States of America; National Cancer Institute, United States of America

## Abstract

The conserved family of cohesin proteins that mediate sister chromatid cohesion requires Scc2, Scc4 for chromatin-association and Eco1/Ctf7 for conversion to a tethering competent state. A popular model, based on the notion that cohesins form huge ring-like structures, is that Scc2, Scc4 function is essential only during G1 such that sister chromatid cohesion results simply from DNA replisome passage through pre-loaded cohesin rings. In such a scenario, cohesin deposition during G1 is temporally uncoupled from Eco1-dependent establishment reactions that occur during S-phase. Chl1 DNA helicase (homolog of human ChlR1/DDX11 and BACH1/BRIP1/FANCJ helicases implicated in Fanconi anemia, breast and ovarian cancer and Warsaw Breakage Syndrome) plays a critical role in sister chromatid cohesion, however, the mechanism through which Chl1 promotes cohesion remains poorly understood. Here, we report that Chl1 promotes Scc2 loading unto DNA such that both Scc2 and cohesin enrichment to chromatin are defective in *chl1* mutant cells. The results further show that both Chl1 expression and chromatin-recruitment are tightly regulated through the cell cycle, peaking during S-phase. Importantly, kinetic ChIP studies reveals that Chl1 is required for Scc2 chromatin-association specifically during S-phase, but not during G1. Despite normal chromatin enrichment of both Scc2 and cohesin during G1, *chl1* mutant cells exhibit severe chromosome segregation and cohesion defects – revealing that G1-loaded cohesins is insufficient to promote cohesion. Based on these findings, we propose a new model wherein S-phase cohesin loading occurs during DNA replication and in concert with both cohesion establishment and chromatin assembly reactions - challenging the notion that DNA replication fork navigates through or around pre-loaded cohesin rings.

## Introduction

The generation of viable cell progeny requires the faithful replication of each parental chromosome, producing identical sister chromatids, and faithful segregation of sister chromatids into daughter cells. Since these two cellular events, DNA replication (S phase) and chromosome segregation (M phase), are temporally separated, cells must maintain the identity of sister chromatids over time - in some cases for decades. Cells achieve this feat through a conserved multimeric protein complex known as cohesins that consist of Smc3, Smc1, Mcd1/Scc1 and Scc3/Irr1 – along side cohesin-bound auxiliary factors Pds5, Rad61/Wapl and metazoan-specific Sororin [1-3]. In addition to their canonical role in sister chromatid tethering, cohesin complexes also function in a multitude of cellular processes including DNA repair, chromatin condensation, transcriptional regulation and rDNA metabolism [4]. The transcription regulatory role may be especially relevant given that mutation in human cohesin subunits (SMC1A/Smc1, SMC3, RAD21/Mcd1/Scc1) and cohesin-regulatory factors (ESCO2/Eco1/Ctf7, HDAC8/Hos1, NPBL/Scc2, APRIN/Pds5, ChlR1/DDX11/Chl1 and BACH1/BRIP/FANCJ/Chl1) result in severe developmental maladies that include Cornelia de Lange Syndrome, Roberts Syndrome, Warsaw Breakage Syndrome and Fanconi Anemia [5-17]. The structure through which cohesins tether together sister chromatids or evoke transcription regulation remains undefined, but models include that DNA is embraced within an SMC lumen, clamped by the folding over of SMC arms to bring head and hinge domains into registration, or sandwiched between SMC head domains capped by Mcd1 (the latter based on crystal structure analyses of the SMC-like Mre11,Rad50,Nbs1 complex [4,18,19]), any of which may assemble into higher order structures [1,4,20-24].

Cohesin binding to chromatin is not sufficient to tether together sister chromatids. Instead, budding yeast Eco1/Ctf7 (herein Eco1), the founding member of a highly conserved family (EFO1/ESCO1 and EFO2/ESCO2 in vertebrates, DECO in flies) of acetyltransferases, is required to convert chromatin-bound cohesins to a tethering competent state [25-30]. To date, cohesin Smc3 is the only known essential Eco1 substrate [31-33]. Eco1 function is essential specifically during S-phase [25,26]. In fact, multiple interactions between Eco1, PCNA (the DNA polymerase ‘sliding clamp’) and Replication Factor C (RFC) complexes that regulate PCNA support the model that Eco1 acetylates Smc3 as sister chromatids emerge from the DNA replication fork [25,31-39]. Contrary to an early report [35], it is now clear that Eco1 binding to PCNA is neither required for Smc3 acetylation nor Eco1 recruitment to DNA [29,39,40]. Thus, critical gaps remain in our understanding of DNA replication-coupled cohesion establishment.

The timing of cohesin association with chromatin appears to profoundly impact the ability of Eco1 to establish cohesion. Supporting DNA replication-coupled cohesion establishment are findings that the essential function of Eco1 maps to S-phase and that Mcd1 expressed after S-phase fails to participate in sister chromatid pairing, although cohesins also associate with DNA before S-phase in both yeast and vertebrate cells [25,26,41-45]. Early cell cycle studies mapped the essential role of the Scc2, Scc4 cohesin deposition complex to S-phase, similar to both Eco1 function and Mcd1 expression [25,26,41,42,46,47]. Biochemical analysis of cohesins as huge ring-like structures, however, led to a popular model that Scc2, Scc4 complex is essential only during G1, enabling replication forks to establish cohesion simply by passing through pre-loaded cohesin rings [48,49]. Subsequent studies support the notion that Scc2, Scc4-dependent cohesin deposition may be required during the G1 portion of the cell cycle, but remain actively debated [22-24,43-45]. Resolving the important issue regarding which temporally-deposited cohesin population in fact participates in cohesion will likely entail analyses of auxiliary factors that promote efficient cohesion establishment.

The DNA helicase Chl1 (and homologs) is of particular interest in that it is crucial for efficient sister chromatid cohesion: *chl1* mutant cells exhibit significant cohesion defects that exceed even essential gene mutations involved in cohesion such as *pol30* (PCNA) and Chl1 is the only helicase thus far shown to associate with Eco1 [35,50-52]. Chl1 is of further import because it is the homolog of both ChlR1/DDX11 and BACH1/BRIP/FANCJ, mutations in which result in Warsaw Breakage Syndrome and both Fanconi anemia and breast and ovarian cancers, respectively [13-15,50,53-62]. In the current study, we show that Chl1 plays a critical role in Scc2 recruitment to chromatin, linking for the first time Scc2 regulation to helicase-dependent alterations of DNA. As important, Chl1 is required for both Scc2 and cohesin recruitment specifically during S-phase, but not G1. Despite normal Scc2 (and cohesin) recruitment to DNA during G1, *chl1* mutant cells exhibit significant cohesion defects. These findings significantly impact current models regarding the temporal coupling of cohesin deposition and cohesin establishment.

## Results

### Chl1 expression and chromatin binding are cell cycle regulated

**Figure 1:**
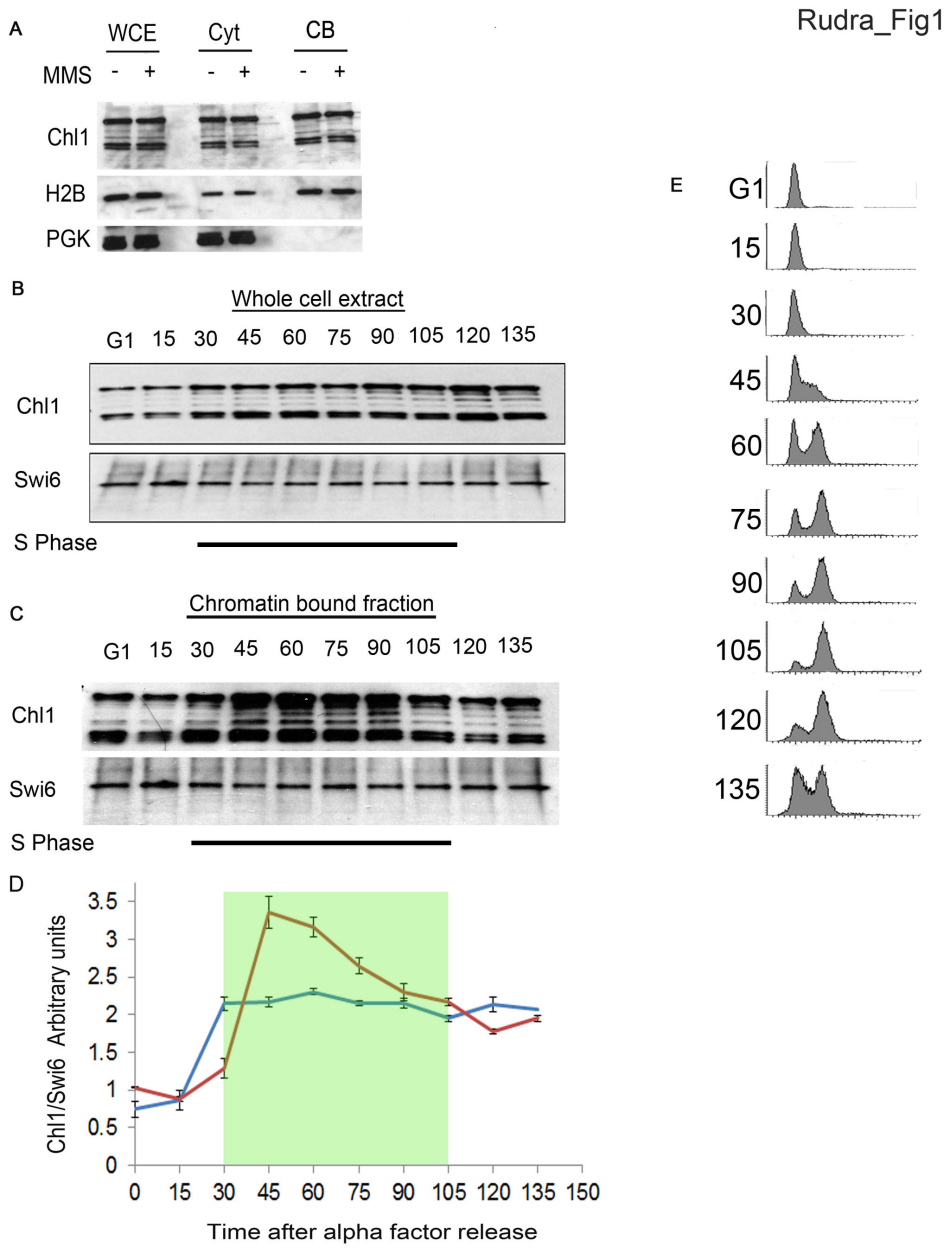
Chl1 expression and chromatin binding are induced during S-phase. A) Logarithmically growing cells expressing Chl1-13Myc (YBS 1129) were harvested and analyzed for chromatin binding with or without MMS exposure. Immunoblots show whole cells extracts (WCE), cytoplasmic fractions (Cyt) and chromatin bound fractions (CB). Histone 2B (H2B) and Phosphoglycerate Kinase (PGK) were probed in parallel as positive controls for chromatin-bound and cytoplasmic proteins, respectively. B) Logarithmically growing cells expressing Chl1-13MYC were synchronized in G1 (alpha factor) and released into fresh medium. Samples were harvested every 15minutes and analyzed by Immunoblotting for Chl1-13MYC and Swi6 as a loading control. Parallel blots were also analyzed for Histone 2B (H2B) and Phosphoglyerinkinase (PGK) to confirm chromatin and cytoplasmic fractions, respectively. C) G1 synchronized cells expressing Chl1-13Myc were released in fresh medium and samples collected every 15 minutes, processed for chromatin binding and probed to detect Chl1-13MYC and Swi6 (loading control). D) Data shown are Chl1 protein levels relative to Swi6 (blue line) and chromatin enrichment relative to Swi6 (red line) over the cell cycle averaged from 3 independent experiments. Shaded portion denotes S-phase. E) Flow cytometric data for cells analyzed in B) and C).

Results from this lab and others suggest that Chl1 is critical for cohesion establishment during S-phase: Chl1 binds Eco1, PCNA and Fen1 and the human homolog ChlR1 stimulates the flap endonuclease FEN-1 involved in both maturation of replication lagging strands and cohesion establishment [35,50,62-64]. Chl1 binding to chromatin thus far, however, has been demonstrated only in response to DNA damage [64], leaving unresolved fundamental issues of Chl1 expression and chromatin recruitment throughout the cell cycle. To address these deficits of knowledge regarding this homolog of clinically-relevant DNA helicases, we first tested the extent to which Chl1 expression is regulated throughout the cell cycle. Logarithmically growing cells expressing epitope-tagged Chl1 were synchronized in G1 (alpha-factor), released into fresh medium to allow for cell cycle progression and samples harvested at 15 minute intervals to assess both cell cycle progression and changes in Chl1 protein levels. The results show that Chl1 is diminished during G1, rises as cells enter S-phase and remains elevated during mitosis (Chl1 typically runs as two bands, most likely due to C-terminal PEST sites [50,65]). In contrast, constitutively expressed Swi6 protein remains unchanged throughout the cell cycle (Figure 1B,D).

If Chl1 promotes cohesion establishment specifically during S-phase, then Chl1 recruitment to chromatin might be similarly reflected in a cell cycle-dependent fashion. To carefully analyze Chl1 recruitment to chromatin, we exploited Triton X-100 cell fractionation assays previously used to demonstrate chromatin-association of a spectrum of factors including Eco1, cohesin, DNA replication initiators and fork stabilization proteins [26,35,66,67]. We validated the cell fractionation procedure using Phosphoglycerokinase (PGK) and Histone 2B (H2B) as cytosolic and chromatin fiduciary markers, respectively. The results show an enrichment of chromatin-associated H2B, and absence of cytosolic component PGK, in the Triton-X-100 insoluble fraction (Figure 1A). Similar to H2B, the bulk of Chl1 resides in the chromatin fraction, although modest levels of both Chl1 and H2B remain soluble. We further tested whether Chl1 chromatin-association might increase in response to DNA damage. Log growth cultures expressing epitope-tagged Chl1 were split and one of the two cultures exposed to 0.1% MMS for 2 hours prior to processing for chromatin binding. The results show that the level of Chl1 chromatin-enrichment is not increased upon MMS treatment, consistent with a model that Chl1 binds chromatin and functions each and every cell cycle (Figure 1A).

Given the critical role for Chl1 in cohesion, it became important to determine whether Chl1 recruitment to chromatin is regulated through the cell cycle. We returned to the validated chromatin-fractionation method described above. The results show that Chl1 recruitment to chromatin increases significantly (>3 fold) as cells enter S-phase (Figure 1C,D). Thus, both Chl1 expression and chromatin recruitment are tightly regulated in normal cycling cells. Notably, while Chl1 protein levels remain elevated during mitosis, Chl1 binding to chromatin decreases as cells exit S-phase - revealing for the first time a post-translational mechanism that drives Chl1 release from DNA. In combination, these results demonstrate that Chl1 expression and DNA binding are tightly regulated through the cell cycle, in support of a model that Chl1 promotes sister chromatid cohesion specifically during S phase and, based on protein interaction studies, in close proximity to the DNA replication fork [35,50,62-64].

### Chl1 regulates cohesion acetylation, but not Eco1 auto-acetylation

**Figure 2:**
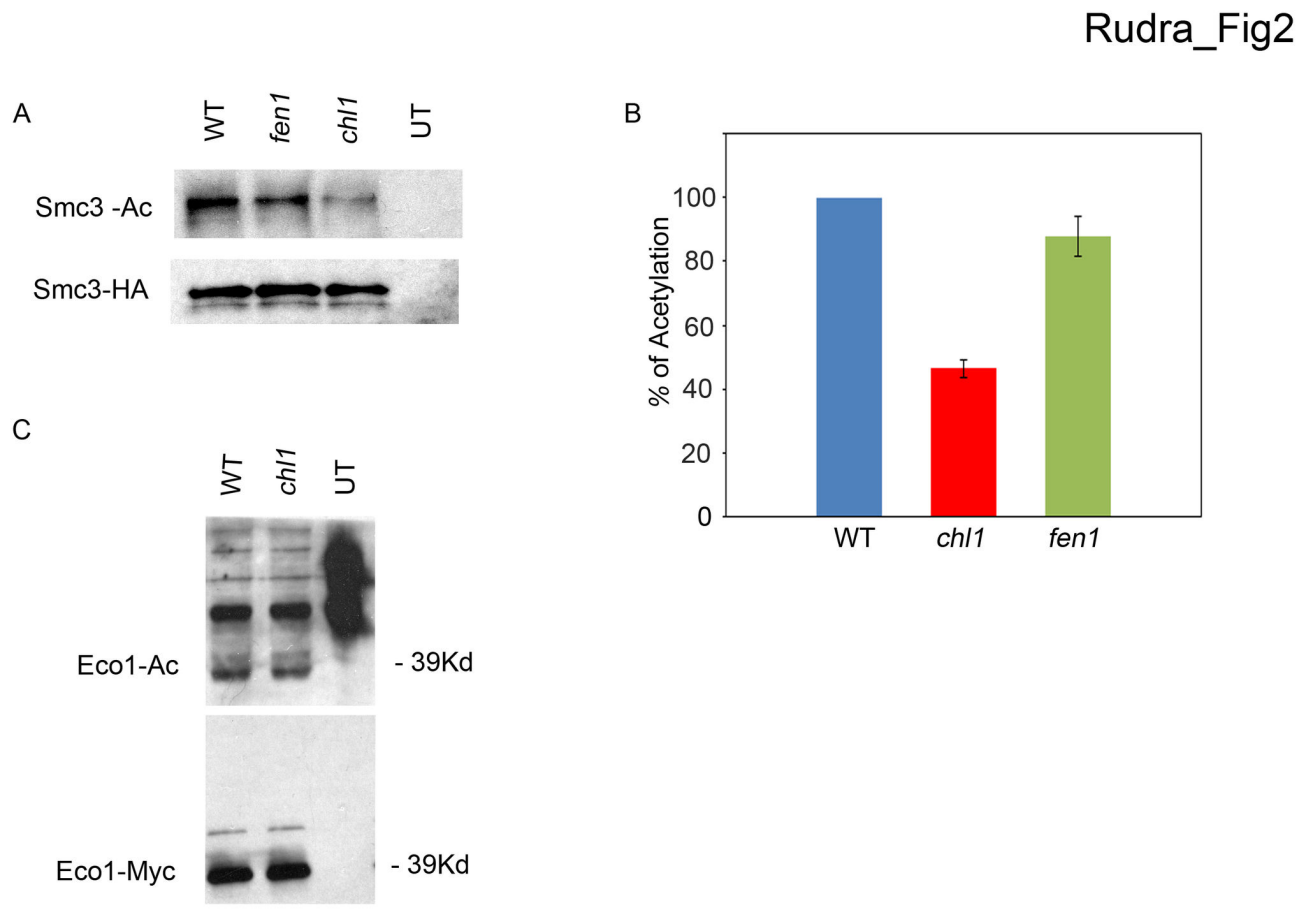
Chl1, but not Fen1, decreases Eco1-mediated Smc3 acetylation without inhibiting Eco1 auto-acetylation. A) Logarithmically growing wild type (YSR 132), *chl1* (YSR 133) and *fen1* (YSR 107) mutant cells expressing Smc3-3HA were harvested and whole cell extracts processed to detect Smc3 and acetylated Smc3. While cell extracts of cells expressing untagged Smc3 also shown. B) Quantification of acetylated Smc3 levels in *chl1* and *fen1* mutant cells compared to that of wildtype cells (normalized to 100%). Data represents the ratio of acetylated Smc3 to total Smc3 levels from three independent experiments. C) Immunoprecipitated Eco1-18MYC from log phase wildtype (YSR 129) and *chl1* (YSR 131) mutant cells probed for total Eco1 (MYC) and auto-acetylated (Ac) levels. Cells expressing untagged Eco1 (UT) (YSR 130) also shown. Data represents the ratio of acetylated Eco1 to total Eco1 levels from three independent experiments.

Presently, the mechanism through which Chl1 promotes efficient sister chromatin cohesion remains unknown. Given that Chl1 is the only helicase shown to interact with the establishment factor Eco1 [50], we hypothesized that Chl1 might regulate Eco1-mediated acetylation of Smc3. We included *fen1* mutant cells in our analysis given that Fen1 also associates with both Eco1 and Chl1 and that both human and yeast cells diminished in Fen1 exhibit cohesion defects [62,63,68]. Logarithmically growing wild type cells and *chl1* and *fen1* single mutant cells, all expressing Smc3-3HA as the sole source of Smc3, were lysed, resulting extracts clarified by centrifugation and incubated with anti-HA-coupled beads. After washing to remove unbound or weakly associated proteins, bead-bound proteins were eluted and analyzed by Western blot. The results show that total levels of Smc3 protein remain unchanged regardless of the presence or absence of either Chl1 or Fen1 (Figure 2A). No signal was obtained for strains expressing untagged Smc3. Querying blots with anti-acetylated lysine antibodies further revealed nearly identical levels of Smc3 modification present in both wildtype and *fen1* mutant cells. In contrast, Smc3 acetylation is markedly decreased in *chl1* mutant cells (Figure 2A and 2B). Thus, Chl1 is critical for Eco1-depedent Smc3 acetylation.

We realized that reduced Smc3 acetylation in *chl1* mutant cells can be explained by at least one of two models: that Chl1 promotes Eco1 acetyltransferase activity or that Chl1 promotes cohesin binding to DNA which in turn becomes a suitable substrate for Eco1. To test the first of these possibilities, we exploited the fact that auto-acetylation is readily detected in Eco1/ESCO proteins [27,29,30,69]. Logarithmically growing wild type and *chl1* mutant cells expressing Eco1-18MYC as the sole source of Eco1 were lysed and the resulting extracts incubated with anti-MYC-coupled beads. After washing to remove unbound or weakly associated proteins, bead-bound proteins were eluted and analyzed by Western blot. Results reveal that total Eco1 protein levels remain unchanged despite the presence or absence of Chl1 (Figure 2C). No signal was obtained for strains expressing untagged Eco1. We also found nearly identical levels of modified Eco1 in wildtype and *chl1* mutant cells using antibody directed against acetylated lysine. These results reveal that Chl1 does not directly regulate Eco1 activity: that decreased Smc3 acetylation in *chl1* mutant cells is not predicated on reduced Eco1 acetyltransferase activity or protein levels.

**Figure 3:**
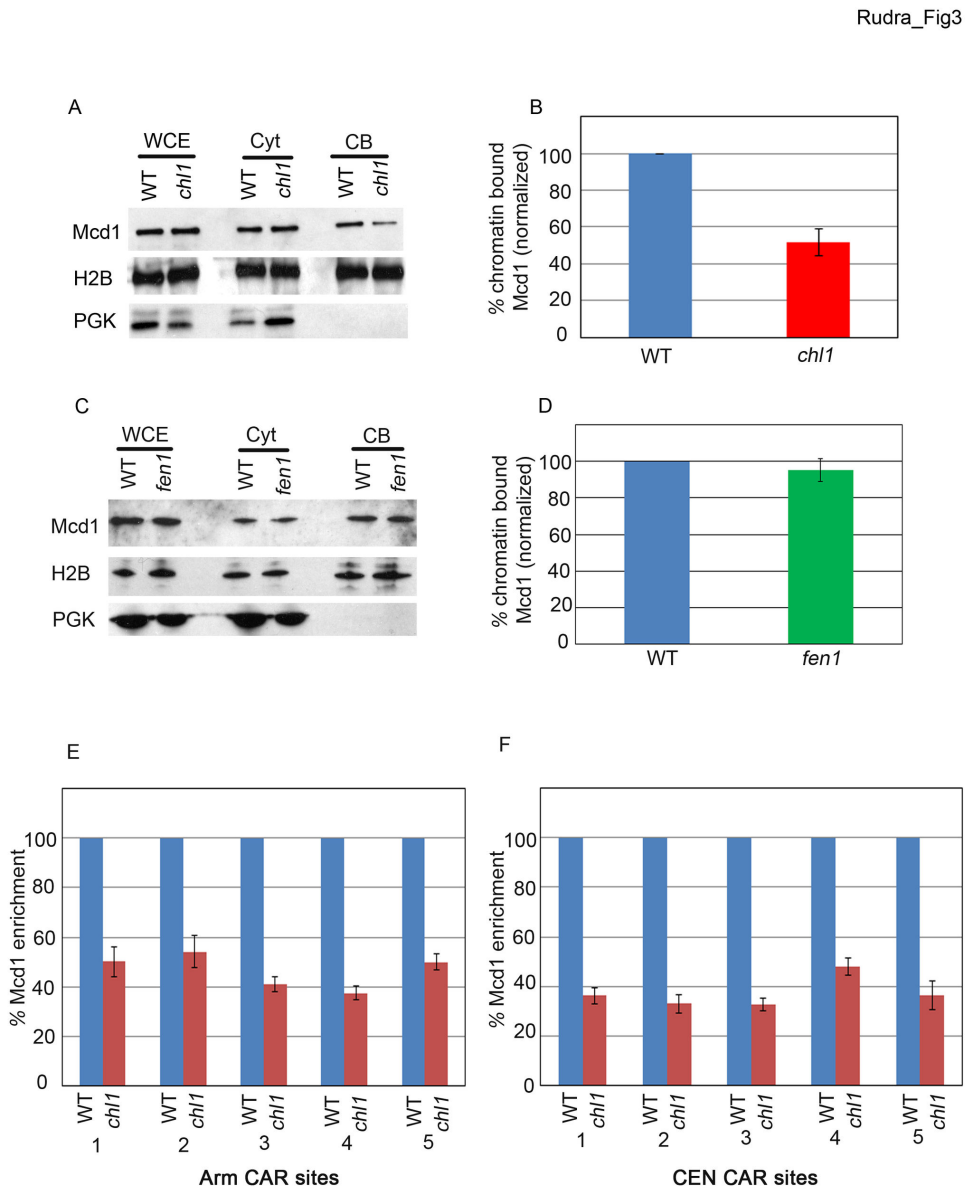
Cells lacking Chl1, but not Fen1, exhibit reduced binding of cohesins to chromatin. A) Logarithmically growing wild type (YBS 1157) and *chl1* (YBS 1175) expressing Mcd1-6HA processed for Mcd1 chromatin binding. Whole cell extracts (WCE), Cytoplasmic fractions (Cyt) and Chromatin bound fractions (CB) shown. Histone 2B (H2B) and Phosphoglycerate kinase (PGK) shown as controls for cytoplasmic and chromatin-bound proteins, respectively. B) Quantification of Mcd1 binding to chromatin in *chl1* mutant cells compared to wildtype levels (normalized to 100%). Mcd1 enrichment to DNA based on the ratio of Mcd1-6HA to Histone 2B levels obtained from 3 independent experiments. C and D) Experimental analysis of *fen1* mutant cells (YSR 117) identical to that shown in A and B for *chl1* mutant cells. E) Enrichment of Mcd1-6HA in *chl1* mutant cells at five independent chromosome arm CAR sites along Chromosome III compared to levels obtained from wild type cells (normalized to 100%). F) Enrichment of Mcd1-6HA as shown in E) except for five centromere (*CEN*) sites.

We next tested the possibility that Chl1 functions in cohesin binding to DNA: that the reduced cohesin acetylation in *chl1* mutant cells is based on loss of cohesin chromatin-association. Log phase wild type, *chl1* and *fen1* single mutant cells expressing Mcd1-6HA as the sole source of this cohesin subunit were lysed and the fraction of chromatin-bound Mcd1 assessed using Triton- X-100 fractionation as described above. Wildtype and *fen1* mutant cells contained nearly identical levels of chromatin-bound Mcd1 (Figure 3C,D). In contrast, *chl1* cells contained a marked reduction (~50% of wildtype levels) of chromatin-bound Mcd1. Importantly, *chl1* mutant cells contained Mcd1 protein levels equivalent to wildtype cells (Figure S1), confirming that the observed reduction of chromatin-associated Mcd1 is due to loss of Chl1 helicase and not reduced Mcd1 expression (Figure 3A,B). To confirm that Chl1 participates in the stable binding of cohesin to DNA, we turned to a chromatin immunoprecipitation (ChIP) strategy that allows for quantification of cohesin enrichment on well-documented cohesin association regions (CARs) [70-72]. We chose 5 independent CAR sites along the arm of chromosome III. Protein-DNA complexes in logarithmically growing wild type and *chl1* mutant cells expressing Mcd1-6HA were cross-linked using formaldehyde, lysed and sonicated to shear the DNA. Chromatin complexes containing Mcd1 were immunoprecipitated, cross-links reversed and DNA amplified using CAR-specific primers. The results show that Mcd1 levels are significantly decreased (about 50% of wildtype levels) for each of the 5 CAR sites (Figure 3E), documenting that Chl1 is critical for stable cohesin binding along chromosome arms.

Cohesin binding at centromeres is uniquely regulated compared to cohesins that associate along chromosome arms: centromeric cohesins occur at elevated levels, along extended regions of DNA and are differentially sensitive to perturbation of cohesin regulators – especially in vertebrate cells [73-78]. Given these unique features, it became crucial to test whether Chl1 also promotes stable binding of cohesin to centromeres. We repeated the ChIP analysis, but this time using 5 centromere (*CEN*) sites within chromosome III, regions well-established as enriched in cohesin binding [71-74]. The results show that *chl1* mutant cells exhibit a significant reduction (<40% of wildtype levels) of cohesin association to centromeres (Figure 3E,F). Further analyses discount the possibility that Mcd1 protein levels are decreased in *chl1* mutant cells (Figure S1A). These findings extend those of prior studies that cohesins are only loosely chromatin-associated in the absence of Chl1/ChlR1 [54,79]. We conclude that Chl1 is critical for stable cohesin-DNA interactions at all CAR sites and that the decrease in cohesin binding in *chl1* mutant cells in part accounts for the reduced levels of Smc3 acetylation (this study).

### Chl1 regulates S phase cohesin binding to chromosomes

**Figure 4:**
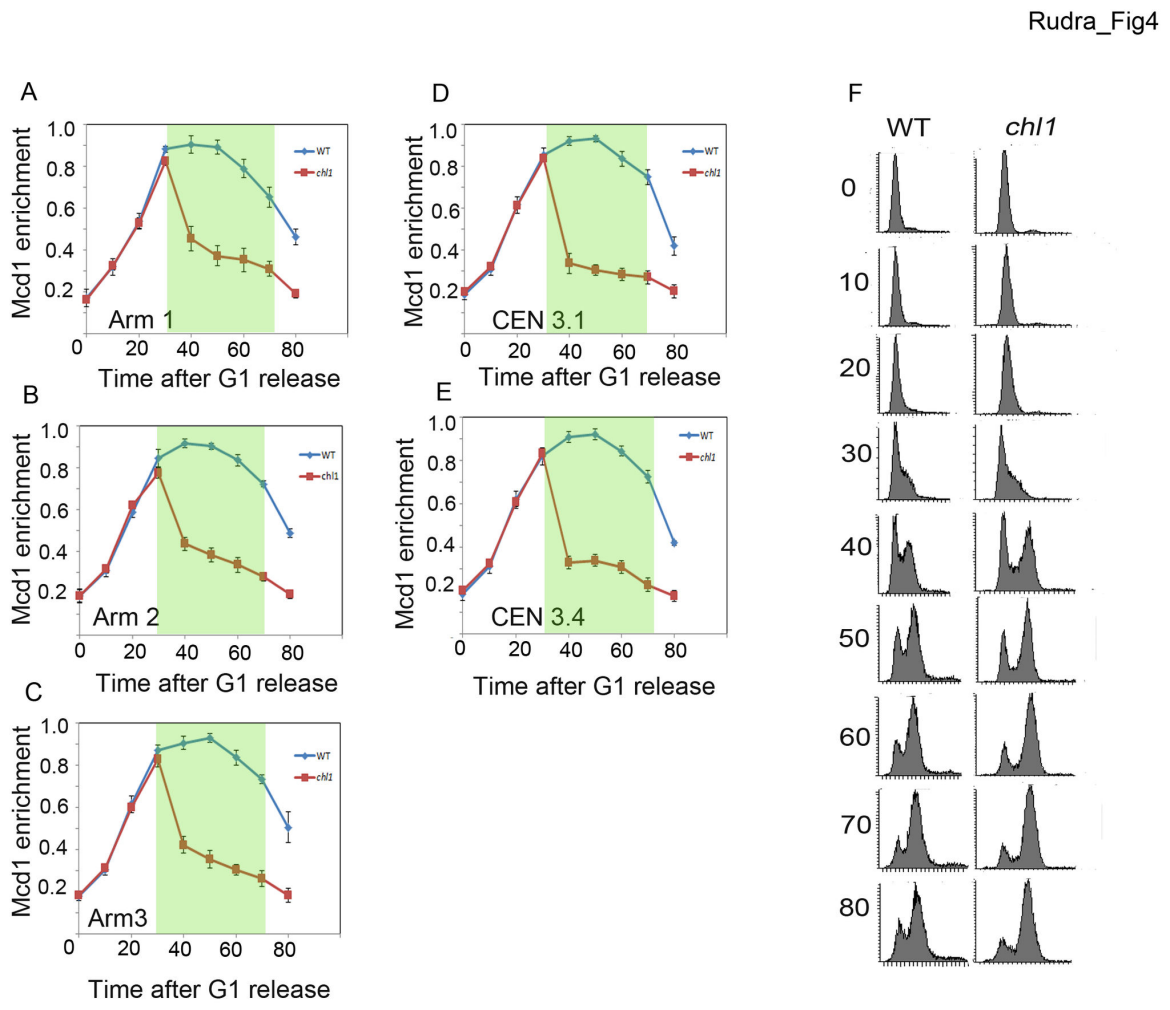
Chl1 regulates cohesin enrichment onto chromosomes during S-phase. A-C) Three panels show Mcd1-6HA binding to three chromosome arm CAR sites in wild type (YBS 1157) (blue lines) and *chl1* mutant cells (YBS 1175) (red lines) progressing through the cell cycle from G1 synchronous release. D, E) Two panels show Mcd1-6HA binding to two unique centromere CAR sites in wild type and *chl1* cells progressing through the cell cycle from G1 synchronous release. F) Flow cytometric data shows DNA content of wild type and *chl1* mutant cells. In all panels, data reflects aliquots harvested at 10-minute intervals. Panels A-E reflect data averaged from three independent experiments. Shaded portion denotes S-phase.

A presumptive but popular model is that cohesin deposition during G1 is required for subsequent sister chromatid tethering reactions that occur during S-phase [23,24]. In contrast is a preceding model that cohesins deposited during S-phase predominantly participate in cohesion establishment [22,80]. Resolving this discrepancy is complicated because *SCC2* mutation abolishes deposition in all phases of the cells cycle such that mapping studies produced conflicting interpretations [39,42,43,72,81]. We realized that Chl1, which promotes cohesion specifically during S-phase and is required for cohesin enrichment onto chromatin, might provide a unique venue from which to address these models. To test whether Chl1 impacts cohesin chromatin-association in a cell cycle-regulated fashion, we repeated the chromatin immunoprecipitations described above on cycling cells released from G1 and harvested at 10 minute intervals so that we could clearly differentiate between G1 versus S-phase cohesin deposition. We queried cohesin enrichment at 5 independent CAR sites: three along chromosome arms and two within the centromere of chromosome III (*CEN3.1* and *CEN3.4*). As expected, cohesin binding to DNA increased during G1 in wildtype cells and this elevated cohesin chromatin-association remained through S-phase, declining only as cells exited mitosis. Notably, cohesin enrichment onto DNA during G1 in *chl1* mutant cells was indistinguishable from that of wildtype cells. Thus, Chl1 is not required to promote stable cohesin association during G1. Importantly, however, cohesin enrichment onto CAR sites dropped precipitously as *chl1* mutant cells entered S-phase (Figure 4A-4E). The decrease in cohesin enrichment failed to recover to wild type levels throughout the remainder of the cell cycle. These findings reveal that cohesin-association with replicated sister chromatids depends on the S-phase activity of Chl1 DNA helicase.

### Chl1 regulates Scc2 chromatin binding to DNA specifically during S-phase

**Figure 5:**
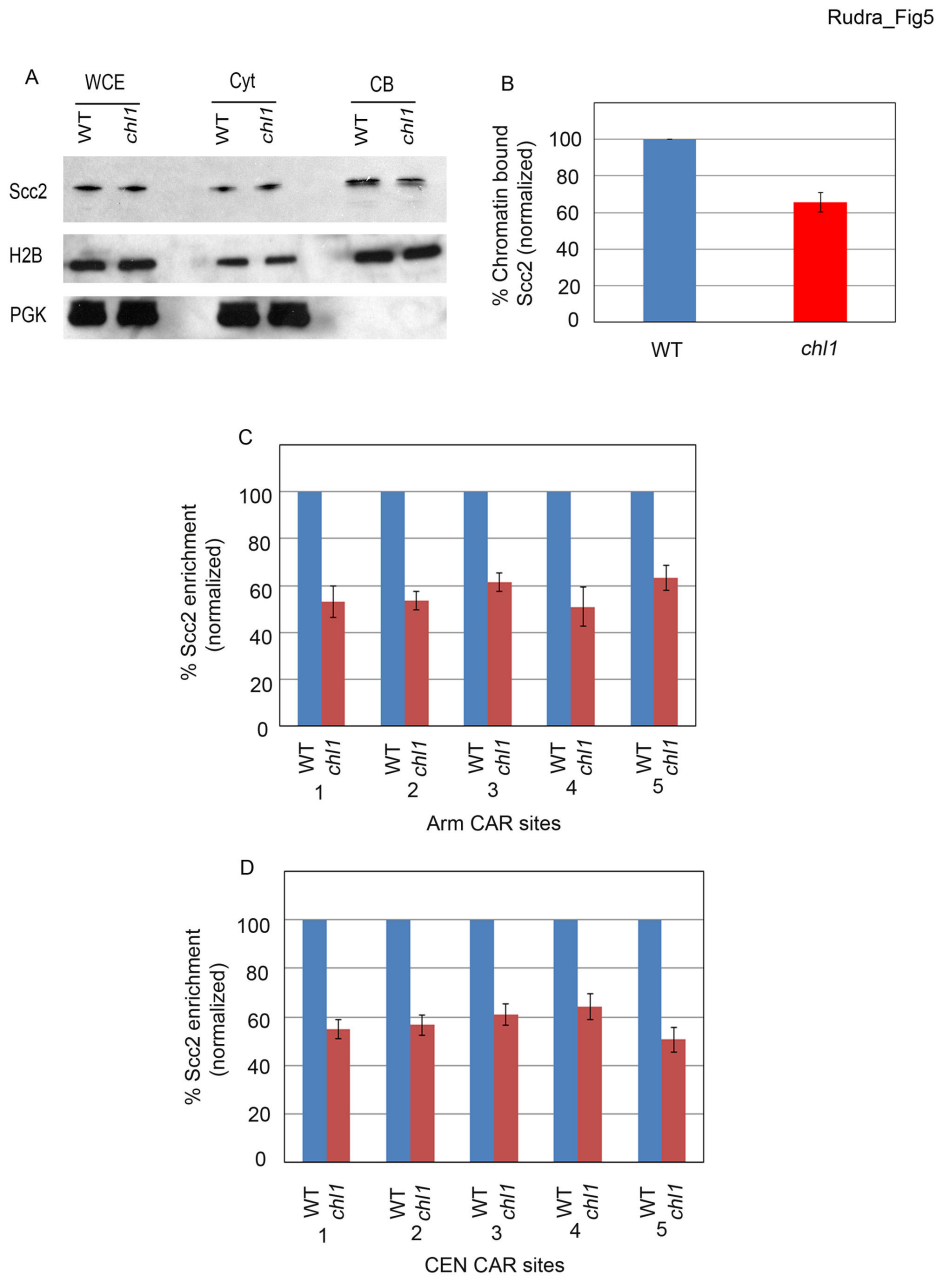
Chl1 regulates the binding of Scc2 to chromatin. A) Logarithmically growing wild type (YSR 135) and *chl1* (YSR 138) expressing Scc2-3HA processed for Scc2 chromatin binding. Whole cell extracts (WCE), Cytoplasmic fractions (Cyt) and Chromatin bound fractions (CB) shown. Histone 2B (H2B) and Phosphoglycerate kinase (PGK) shown as controls for cytoplasmic and chromatin-bound proteins, respectively. B) Quantification of Scc2 binding to chromatin in *chl1* mutant cells compared to wildtype levels (normalized to 100%). Scc2-3HA enrichment calculated from 3 independent experiments. C) Enrichment of Scc3-3HA in *chl1* mutant cells at five independent chromosome arm CAR sites along Chromosome III compared to levels obtained from wild type cells (normalized to 100%). D) Enrichment of Scc2-3HA as shown in C) except for five sites that map across centromere III (*CEN*).

The above studies reveal that *chl1* mutant cells are deficient in cohesin enrichment onto DNA specifically during S-phase, a deficit that leads to cohesion loss. However, the mechanism through which Chl1 promotes cohesin enrichment remains unknown. We hypothesized that Chl1 might be required for efficient recruitment of the Scc2, Scc4 cohesin-deposition complex to DNA. To test this possibility, log phase wild type and *chl1* mutant cells expressing Scc2-3HA as the sole source of Scc2 function were lysed and the fraction of chromatin-bound Scc2 assessed using Triton X-100 fractionation as described above. Compared to wildtype cells, *chl1* mutant cells contained a marked reduction (~40% of wildtype levels) of chromatin-bound Scc2. Whole cell extracts from wildtype and *chl1* mutant strains contained identical Scc2 levels, confirming that the reduction of Scc2 binding was due to loss of Chl1 helicase and not altered Scc2 expression (Figure 5A,B and Figure S1B). Is Scc2 binding to DNA reduced specifically at CAR sites? We investigated whether Chl1 participates in the stable binding of Scc2 at five independent CAR sites that reside along the arm of chromosome III and also five CAR sites within *CEN3* using chromatin immunoprecipitations. The results show that Scc2 levels are significantly decreased (50-60% of wildtype levels) for each of the ten CAR sites queried, revealing for the first time that Chl1 is a critical regulator of Scc2 enrichment onto DNA (Figure 5C,D).

**Figure 6:**
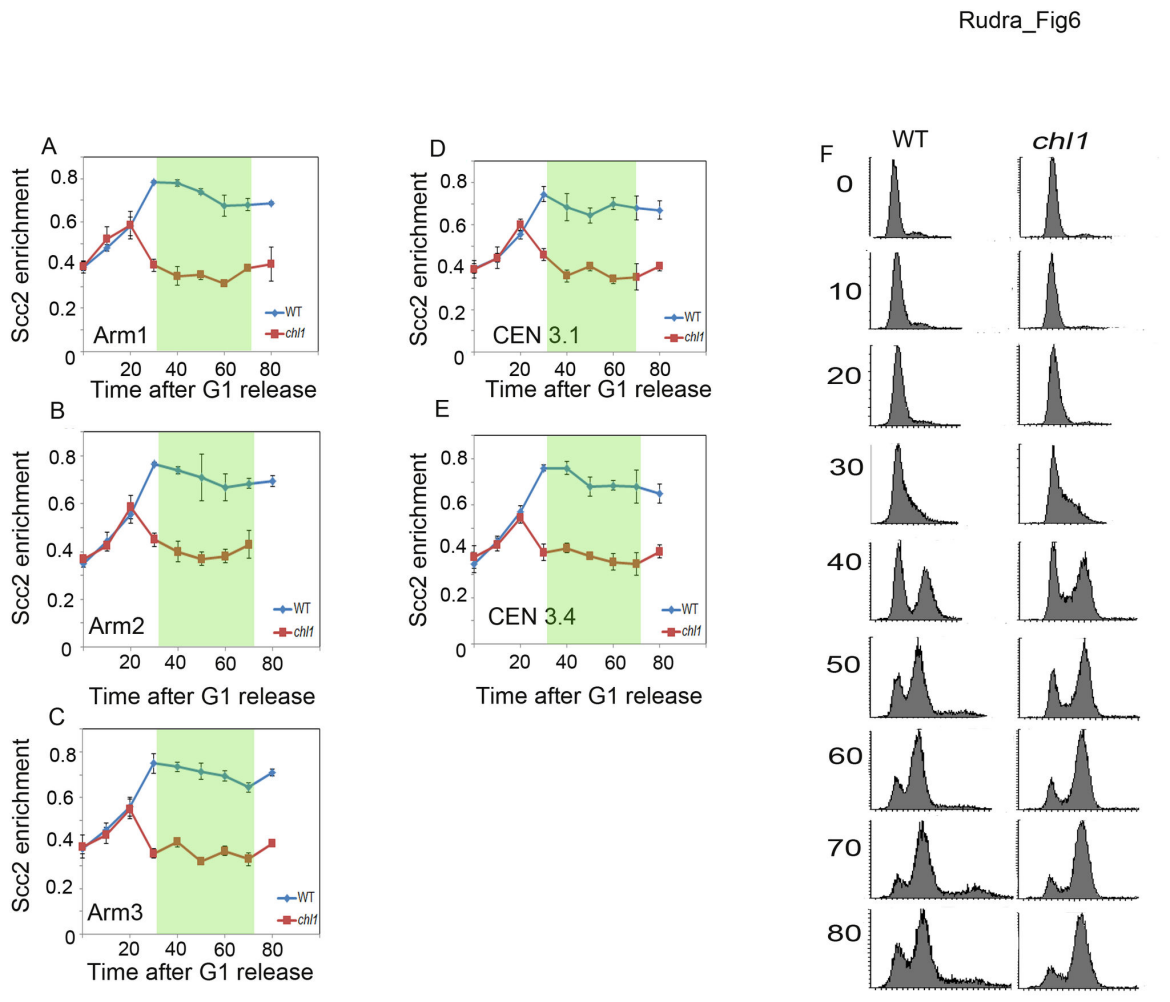
Chl1 regulates Scc2 binding onto chromosomes specifically during S-phase. A-C) Three panels show Scc2-3HA binding to three chromosome arm CAR sites in wild type (YSR 135) (blue lines) and *chl1* mutant cells (YSR 138) (red lines) progressing through the cell cycle from G1 synchronous release. D, E) Two panels show Scc2-3HA binding onto two unique centromere CAR sites in wild type and *chl1* cells progressing through the cell cycle from G1 synchronous release. F) Flow cytometric data shows DNA content of wild type and *chl1* mutant cells. In all panels, data reflects aliquots harvested at 10-minute intervals. Panels A-E reflect data averaged from three independent experiments. Shaded portion denotes S-phase.

If we are correct that Chl1 is critical for the enrichment of cohesin to DNA specifically during S-phase, then *chl1* mutant cells should similarly exhibit loss of Scc2 binding to DNA as cells enter S-phase, but not before. To test this prediction, we performed chromatin immunoprecipitation assays taking samples at 10 minute intervals from synchronized wild type and *chl1* mutant cells expressing Scc2-3HA. The results show that Scc2 enrichment at all five CAR sites (three arm and two centromere sites) prior to S-phase is identical in both wildtype and *chl1* mutant cells. Wildtype cells continued to recruit Scc2 throughout S-phase and maintained this level into mitosis. As soon as *chl1* mutant cells entered S-phase, however, the level of Scc2 binding to DNA dropped significantly at all CAR sites tested and remained low even after S-phase (Figure 6). In combination, these results document that Chl1 is critical for the stable association of Scc2 with DNA during S-phase and that this DNA replication-coupled deposition is critical for sister chromatid cohesion establishment.

## Discussion

### Cohesins that participate in cohesion become chromatin-associated during S-phase

**Figure 7:**
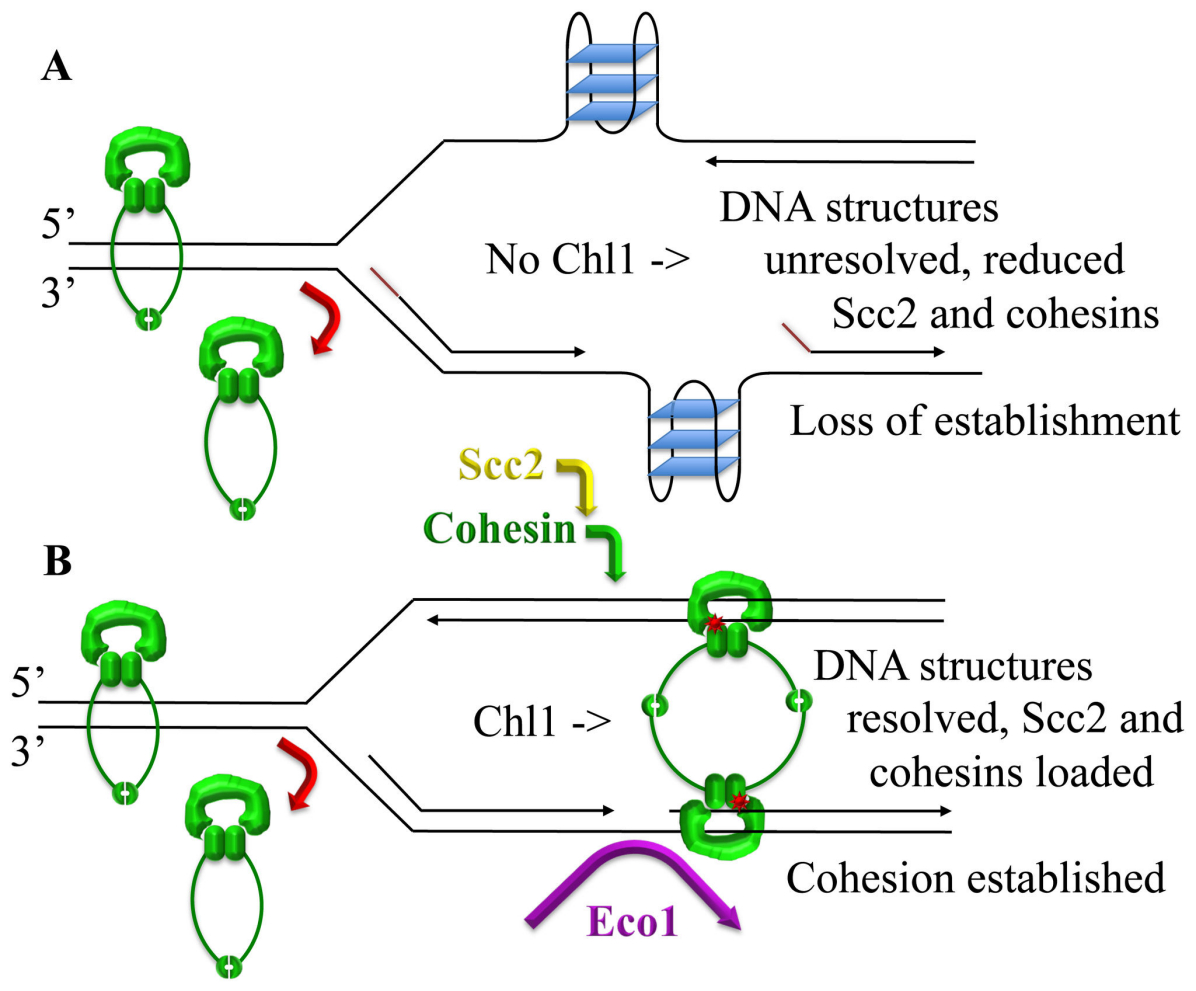
Model regarding Chl1 role during cohesion establishment. A) Cohesins (green) that associate with DNA during G1 are highly dynamic. During S-phase, these G1-loaded cohesins are bumped off (red arrow) by the DNA replication fork (not shown) and fail to participate in cohesion establishment. In the absence of Chl1 DNA helicase, secondary DNA structures (forked structures with RNA primers in red; G-quadruplexes or G4 in blue with looped DNA) form immediately behind the DNA replication fork and preclude the stable association to DNA of both Scc2 and cohesin – leading to loss of sister chromatid cohesion. B) In the presence of Chl1 DNA helicase, secondary DNA structures are resolved and allow for recruitment of both Scc2 (yellow arrow) and cohesin (green arrow). Cohesin recruitment specifically during S-phase, and subsequent acetylation (red) by Eco1 (purple arrow) during S-phase, result in establishment of sister chromatid tethering. Speculative conformation shown of cohesin-association to DNA through Mcd1 capping of SMC complex (reviewed in [4]), that differentiates stable cohesin-binding from the highly labile cohesin association that occurs during G1 (see text for details).

The issue regarding the population of DNA-associated cohesins which both serve as Eco1 substrates and participate in sister chromatin-tethering reactions remains a critical but enigmatic topic of cell biology. One of the major revelations of the current study involving Chl1 is that cohesins that associate with DNA during G1 fail to produce sister chromatid cohesion: *chl1* mutant cells load cohesins onto DNA during G1 to levels identical to wildtype cells and to appropriate CAR sites - yet exhibit significant cohesion defects. While *chl1* mutants are viable, the resulting cohesion defect (35%) nearly rivals that of many cohesin mutants (50-65%) and exceeds that of essential cohesion gene mutations such as *pol30/pcna* (20%) [40]. We also note that loss of cell viability does not necessarily equate to cohesion defects in budding yeast; nor do cohesion defects necessarily impact proper chromosome segregation [82]. It is not, however, the level of cohesion defect that occurs in *chl1* mutant cells that is of interest here, but the cell cycle specificity that provides a unique tool from which to assess when cohesin deposition is required for establishment. What then is the basis of the cohesion defect in *chl1* mutant cells? As opposed to the normal cohesin enrichment onto DNA that occurs during G1, *chl1* mutant cells exhibit dramatic defects in cohesin enrichment specifically during S-phase. These findings document that cohesin must associate with DNA during S-phase to both serve as an Eco1 substrate and participate in cohesion (Figure 7). This re-emerging view, which we term *Replication-coupled cohesion deposition*, is supported by numerous findings including that 1) Eco1 can acetylate cohesins prior to S-phase, but that cohesins acetylated during G1 fail to produce sister chromatin cohesion and 2) Scc2 function is essential predominantly during S-phase, even though deposition occurs throughout other portions of the cell cycle [39,42-45,72,80].

Our *Replication-coupled cohesion deposition* model contrasts popular views that cohesin deposition onto DNA is required during G1 – a notion arising not from analysis of deposition complexes but instead from biochemical studies that cohesins might form a huge ring-like complex [48,49]. The rationale was as follows: if cohesins form huge rings and become loaded prior to DNA replication, then establishment might simply occur by passage of the DNA replication fork through cohesin rings. This *Replication-through-a-ring* model quickly gained popularity and to this day remains widely-discussed [23,24], despite being contrary to prior results that Scc2, Scc4 function is required during S-phase and that *eco1* mutant cells exhibit cohesion defects despite normal cohesin deposition and subsequent DNA replication [25,26,36]. Partly in response to these challenges, analyses of Scc2, Scc4 (Mis4,Ssl3 in fission yeast) was re-visited, with the results suggesting instead that deposition is critical during G1 [43-45]. The conflicting interpretations and ongoing debate may be understandable given that i) Scc2, Scc4 is required for all cohesin deposition, ii) deposition occurs throughout the cell cycle, iii) conditional alleles can become refractile to inactivation once complexed with other proteins and iv) very little cohesin (13%) is required to maintain sister chromatid cohesion [77]. With regards to this latter point, low levels of cohesins that associate with early-replicating domains may contribute to underestimating the importance of S-phase cohesin deposition in cell cycle mapping studies. Ultimately, size estimates indicating that the DNA replisome (including leading and lagging strand polymerases, helicase/primase, and extruded DNA loops or ‘trombones’ that occur during lagging strand synthesis) is larger than the lumen posited to form upon Smc1,3 and Mcd1 assembly provided a convincing argument against a simplistic *Replication-through-a-ring* [83]. In fact, the cohesin lumen is likely smaller than first posited given biochemical and FRET studies that Mcd1 sits atop Smc1,3 heads and does not necessarily participate in lumen formation per se [84].

In response, the *Replication-through-a-ring* model evolved in one of two interesting ways. The first of these involved *Replication fork relaxation*. Here, the notion forwarded was that the replisome partially disassembles upon encountering cohesins such that the extruded DNA loop or ‘trombone’ that occurs during lagging strand synthesis collapses – allowing for a more streamlined or linearized replisome capable of passing through pre-loaded cohesin rings [83]. If true, then *Replication fork relaxation* would occur over a 1,000 times per yeast genome replication to accommodate cohesin-bound loci that appear on average every 12 kb and amidst a million iterations of lagging strand processing [70,71,85]. A second branch through which the *Replication-through-a-ring* model evolved included cohesin ring opening/re-closing reactions. In this *Cohesin dynamics* model, opening of G1-loaded cohesins during S-phase was posited to allow for the migration of fully intact replisomes around an open ring – fork relaxation is not required [45]. It remains unclear how cohesin rings, deposited during G1, open and yet remain bound to single-stranded DNA even as the DNA templates for new synthesis. While there is limited evidence that replication fork stability factors associate with cohesins in vitro [86,87], additional challenges (how does DNA polymerase access ssDNA bound by cohesin; how does the sliding clamp PCNA navigate around cohesin-bound DNA, etc) provide little relief for models that rely on G1-loaded cohesins.

An important feature of the *Replication-coupled cohesion deposition* model posited here is that replisome relaxation and cohesin ring gymnastics, requirements imposed by G1-loaded cohesin rings for replisome progression, are obviated. What happens to these pre-replicative cohesins? The data from the current study suggests that Scc2 and cohesins loaded during G1 are normally bumped off upon cell entry into S-phase (Figure 7). Replisome bump-off (whether through fork progression or formation of secondary DNA structures that arise upon replication fork passage) is consistent with evidence that cohesins that associate with DNA during G1 are highly dynamic and cycle between soluble pools and weakly bound chromatin complexes [86,88-90]. Alternatively, the decrease in Scc2 and cohesin enrichment in *chl1* mutant cells might reflect altered kinetics in which formation of secondary DNA structures precludes only new deposition during S-phase while dissociation rates remain unaffected. Discerning between these two possibilities, or some combination thereof, awaits further studies. In the meantime, an additional feature of the *Replication-coupled deposition* model is that cohesin enrichment occurs during S-phase when both sister chromatids are present (Figure 7). This model lends support to a dimer/oligomer-based mechanism of sister chromatid tethering and maintenance [4,77].

### Chromatin structures that form during DNA replication impact recruitment of Scc2

The timing, and thus position relative to the DNA replication fork, through which Scc2 and cohesin are both recruited to chromatin and participate in establishment is likely one of the most significant informers of cohesin function. Thus, the second major revelation of the current study is that Scc2, and in turn cohesin, binding to chromatin is dependent on Chl1. How does Chl1 promote Scc2 recruitment to chromatin? To date, the only known role for Chl1 (including homologs ChlR1 and BACH/FANCJ) is to bind and resolve secondary DNA structures such as forks and flaps (that arise during Okazaki maturation) and G quadruplex (G4) substrates [54,55,62,91-93]: guanine-rich motifs that form 4-stranded coplanar structures posited to form upon DNA replisome passage [94,95]. The role of Chl1 homologs in resolving G4s may be particularly informative in that roughly 50% of the predicted 350,000 structures reside at replication origins in humans and likely form immediately behind the DNA replication fork on exposed ssDNA. G4s are capable both of impacting protein recruitment to DNA (including histones) and likely are critical regulators of transcription regulation [94,95]. These G4 attributes nicely align with findings that Chl1/ChlR1 is critical for maintenance of heterochromatin, tissue development, and regulates protein-associations with DNA including HPV and the epigenetic factor HP1α [4,13,53,54,96,97].

The combination of these reports suggests that secondary DNA structures (including forked substrates and G4 structures) that arise during DNA replication are capable of prohibiting Scc2 enrichment to DNA, which in turn precludes both cohesin deposition and cohesion establishment (Figure 7). The positioning of Chl1 helicase to replicating/maturing sister chromatids, and apparent role of chromatin structure in Scc2/cohesin binding to DNA, supports an emerging view of cohesins in chromosome condensation. Despite early evidence that mutations in either *ECO1, MCD1 or PDS5* produce severe chromosome condensation defects [25,46,98], the role of cohesins in chromatin architecture remains largely underexplored. More recent evidence that *RAD61/WAPL* mutations, originally thought to rescue cohesin mutation cohesion defects [99,100], instead rescues condensation defects produced by cohesin mutations, dramatically altered the landscape regarding cohesin biology [82]. While seldom articulated, we note that cohesin mutations also appear to alter chromatin compaction in cells of vertebrate models used to recapitulate phenotypes observed in human developmental disorders such as Cornelia de Lange Syndrome and Roberts Syndrome [101,102].

There is wide-spread support for a model that Scc2 and cohesin deposition not only occur during chromatinizing reactions, but that chromatin remodeling complexes play reciprocal roles in cohesin binding to DNA [2,103-105]. Thus, cohesin deposition and activation not only promote cohesion/condensation, but occur in a context through which other chromatin modifications arise [4]. Given the role of Chl1 homolog mutations in developmental disorders like Warsaw breakage syndrome, Fanconi anemia, breast and ovarian cancers [106], assessing the role of Chl1 in chromatin architecture from patient cells may provide important insights regarding the mechanisms through which these maladies arise.

### Chl1 DNA helicase expression and chromatin recruitment are tightly regulated throughout the cell cycle

Chl1 is the homolog of human ChlR1/DDX11 and BACH1/BRIP/FANCJ helicases - thus the paucity of information regarding Chl1 expression, chromatin recruitment and regulation is surprising. During the final stages of manuscript preparation, a report regarding Chl1 was published by the Uhlmann lab [107]. That study, similar to ours, confirmed a role for Chl1/ChlR1 in stable association of cohesin to chromatin and that cohesin acetylation was reduced in *chl1* mutant cells ( [54,79,107] and current study). Surprisingly, however, few other similarities with respect to Chl1 regulation exist between that and the current study. For instance, our results clearly reveal that Chl1 binding to chromatin rises dramatically as cells enter S-phase and fall precipitously as cells exit S-phase - even in the continued accumulation of Chl1 protein levels. These results are comparable to those obtained for ChlR1: expression peaks in proliferating cells and mirrors that of PCNA [62]. In contrast, Borges and colleagues failed to detect cell cycle regulation of Chl1 in terms of either expression or chromatin recruitment [107]. We have yet to resolve the difference results obtained between the two studies, but note that Chl1 chromatin-association was performed in cells held for hours in an arrested state [107] - not in naturally cycling cells as performed in the current study. These findings support our conclusion that Chl1 recruitment to DNA is promoted by active DNA replication. We further note intriguing results that Chl1 is required for Ctf4 (Pol-αlpha-binding factor that promotes cohesion [108,109]) to bind DNA specifically during S-phase and not other portions of the cell cycle [107]. Thus, at least the fact that Chl1 exhibits S-phase specific activities does not appear to be in dispute. Given our results from chromatin immunoprecipitation studies that Chl1 is critical for Scc2 and cohesin recruitment to DNA specifically during S-phase (but not G1), we conclude that Chl1 expression and recruitment to chromatin are tightly cell cycle regulated.

## Experimental Procedures

### Media and strains

*Saccharomyces cerevisiae* strains and growth media are as described in reference [50] and listed in Table 1. Strain constructions and primer sequences are included as File S1.

**Table 1:** Strains used in this study.

Strains	Genotype
YBS 1019	*MATa ade2-101 his3∆200 leu2 lys2-801 trp1∆63 ura3-52*
YBS 1020	*MATalpha ade2-101 his3∆200 leu2 lys2-801 trp1∆63 ura3-52*
YBS 1129	*MATa ade2-101 his3∆200 leu2 lys2-801 trp1∆63 ura3-52 CHL1:13Myc:URA3*
YBS 1157	*MATa ade2-101 his3∆200 leu2 lys2-801 trp1∆63 ura3-52 MCD1:6HA:TRP1*
YBS 1175	*MATa ade2-101 his3∆200 leu2 lys2-801 trp1∆63 ura3-52 SCC1:6HA:TRP1CHL1::KAN^r^*
YSR 132	*MATa ade2-101 his3∆200 leu2 lys2-801 trp1∆63 ura3-52 SMC3:3HA:TRP1*
YSR 133	*MATa ade2-101 his3∆200 leu2 lys2-801 trp1∆63 ura3-52 SMC3:3HA:TRP1 CHL1::HIS3*
YSR 107	*MATa ade2-101 his3∆200 leu2 lys2-801 trp1∆63 ura3-52 SMC3:3HA:TRP1 FEN1::HIS3*
YSR 129	*MATa ade2-101 his3∆200 leu2 lys2-801 trp1∆63 ura3-52 ECO1::HIS3 ECO1:18MYC:LEU2*
YSR 130	*MATa ade2-101 his3∆200 leu2 lys2-801 trp1∆63 ura3-52 ECO1::HIS3 ECO1:LEU2*
YSR 131	*MATa ade2-101 his3∆200 leu2 lys2-801 trp1∆63 ura3-52 ECO1::HIS3 ECO1:18MYC:LEU2 CHL1::TRP1*
YSR 117	*MATa ade2-101 his3∆200 leu2 lys2-801 trp1∆63 ura3-52 MCD1:6HA:TRP1 FEN1::KAN^r^*
*YSR 135	*MATa ade2-1 his3-11,15 leu2-3,112 trp1-1 ura3-1 SCC2:3HA:TRP1*
*YSR 138	*MATa ade2-1 his3-11,15 leu2-3,112 trp1-1 ura3-1 SCC2:3HA:TRP1 CHL1::HIS3*

All strains are of S288C background except where noted (* are W303 strains).

### Chromatin binding assay

Logarithmic growing cells were harvested and processed for chromatin binding assay as previously described [35] with the following modifications. Briefly, culture densities were normalized (0.4-0.5 OD_600_, 50ml) and harvested in 25ml CB1 buffer (50mM Sodium citrate, 40mM EDTA, 1.2M sorbitol, pH 7.4), washed with distilled water and 25ml 1.2M sorbitol. The cells were pelleted by centrifugation (1800 rpms for 5 minutes) and resuspended in 1.125ml of CB1 buffer to which was added 125ul of spheroplast mix (125ul CB1, 50ul zymolase, 5ul BME) and then incubated with gentle shaking for 1 hour at room temperature. Spheroplast efficiency was monitored thereafter every 10 minutes until 95% cell lysis was achieved upon exposure to 10% SDS. Spheroplasts were washed 2X with 1.2M sorbitol, resuspended in 425ul of 1.2M sorbitol and snap-frozen in liquid N2. Frozen samples were supplemented with protease inhibitor cocktail (Sigma), thawed on ice and 50ul lysis buffer (500mM Lithium acetate, 20mM MgSO4, 200mM HEPES, pH 7.9) and 20ul of 25% Triton-X-100 added and gently mixed. Cell lysis was monitored by microscopy to achieve 90-95% lysis. WCE fractions were collected and solubilized with 2X Laemmli buffer (Sigma). The remaining lysate was centrifuged at 12,000g for 15 minutes. Supernatant consisting of non-chromatin bound fraction was collected and solubilized with 2X Laemmli buffer (Sigma). The pellet was re-suspended in Lysis buffer + 150mM NaCl and centrifuged at 12,000g for 15 minutes. 2 Units of DNase I (Roche), 5mM MgSO4, and protease inhibitor cocktail was added to the resuspended pellet and incubated at 4°C for 1 hour to release chromatin-bound proteins. The resulting sample was centrifuged at 14,000g for 5 minutes and the supernatant containing released chromatin-bound proteins solubilized with 2X Laemmli buffer (Sigma). Whole cell extract, cytoplasmic and chromatin bound fractions were resolved by SDS-PAGE electrophoresis and analyzed by Western blot using anti-MYC 9E10 (1:1000) (Santa Cruz), anti-HA (1:500) (f7) in combination with goat anti mouse HRP (1:10,000) (Bio-Rad) or by anti-Histone 2B (1:2000) (Santa Cruz) in combination with goat anti Rabbit HRP (1:10,000) or by anti-phosphoglycerate kinase (Invitrogen) in combination with goat anti mouse HRP (1:10,000) (Bio-Rad) and ECL plus (GE healthcare) for visualization.

### Acetylation assay

Cells were processed for acetylation assays as described [32] with the following modifications. Briefly, logarithmically growing cells expressing Smc3-3HA were harvested, suspended in IPH150 buffer (50mM TRIS, 150mM NaCl, 5mM EDTA, 0,5% IGEPAL, 10mM Sodium Butyrate, 1mM DTT, pH8) and protease inhibitor cocktails (Sigma). 500μl of glass beads (Biospec) were added and cells snap-frozen in liquid nitrogen. Cells were then thawed on ice, lysed using mechanical lysis (Biospec mini bead Beater), briefly centrifuged and clarified extract incubated overnight with EZ view anti-HA affinity gel (Sigma). The bead-protein complexes were washed with IPH50 buffer (50mM TRIS, 50mM NaCl, 5mM EDTA, 0.5% IGEPAL, 10mM Sodium Butyrate, 1mM DTT, pH8) to remove unbound or weakly-associated proteins prior to centrifugation at 10,500rpms (TOMY). The bead-bound proteins were solubilized with 2X Laemmli buffer (Sigma) and analyzed by SDS-PAGE Western blot using anti-HA (1:2000) (F7, Santa Cruz) in combination with goat anti mouse HRP (1:10,000) (Bio-Rad) or anti-acetylated Lysine antibody (1:2000) (ST1027, Calbiochem) in combination with goat anti Rabbit HRP (1:15,000) and ECL-Prime (GE Healthcare).

### Chromatin Immunoprecipitation and CHIP primers

Cells were processed for chromatin immunoprecipitation as described [72] with the following modifications. Cells expressing Scc1-6HA were treated with 1% formaldehyde for 2 hours at room temperature to crosslink protein-DNA complexes. Cells were then harvested by centrifugation and resuspended in HEPES/Sorbitol buffer (20mM HEPES, 1.2M Sorbitol, 0.5mM PMSF, 2mg Zymolase (Seikagaku)) and incubated at 30°C for 30 minutes to spheroplast cells. Spheroplasts were washed several times and resuspended in Lysis buffer (1% SDS, 10mM EDTA, 0.5mM EGTA, 10mM HEPES, protease inhibitor cocktail). Lysed cells were sonicated on ice for 6 cycles of 10 seconds. The suspension was centrifuged at 15000 rpm (TOMY) at 4°C and the supernatant diluted with IP buffer (0.01% SDS, 1.1% Triton-X-100, 1.2mM EDTA, 16.7mM TRIS, pH 8.1, 167mM NaCl). The suspension was then centrifuged at 8400g for 10 minutes and the supernatant collected as the chromatin solution. The chromatin solution was incubated with anti-HA EZ view affinity gel (Sigma) overnight, protein-bound bead complexes washed with TSE 150 buffer (0.1% SDS, 1% Triton-X-100, 2mM EDTA, 20mM TRIS-HCl, 150mM NaCl, pH 8.1) and formaldehyde crosslinks reversed by incubating with 5M NaCl at 65°C for 4 hours. DNA from the resulting sample was extracted using Phenol-Chloroform-Isoamyl alcohol, precipitated with EtOH and resuspended. Extracted DNAs were amplified by PCR prior to analysis by Agarose gel electrophoresis (1.5% agarose). Chromatin enrichment was quantified in the following manner. Band intensities from DNA gels were obtained using Photoshop CS5. To calculate enrichment for each CAR site within each strain, ChiP band intensity (minus background intensity obtained from GST non-specific control) was divided by input intensity (minus background intensity obtained from GST non-specific control) for each time point. The resulting *chl1*/wildtype ChIP ratios shown represent averaged data obtained from three independent experiments. Primer co-ordinates for the chosen CAR sites on chromosome III are as follows: Arm 1 (Primer pairs DK-EU-25 and DK-EU-26, SGD co-ordinates 194137 to 194479), Arm 2 (Primer pairs DK-EU-29 and DK-EU-29 and DK-EU-30, SGD co-ordinates 195996 to 196386), Arm 3 (Primer pairs DK-EU-33 and DK-EU-34, SGD co-ordinates 198380 to 198762), Arm 4 (Primer pairs *MAT36F* and *MAT36R*, SGD co-ordinates 191257 to 191599), Arm 5 (Primer pairs *MAT37F* and *MAT37R*, SGD co-ordinates 191778 to 192108). *CEN1* (Primer pairs *CEN3L5F* and *CEN3L5R*, SGD co-ordinates 99171 to 99460), *CEN2* (Primer pairs *CEN3L3F* and *CEN3L3R*, SGD co-ordinates 108724 to 109020), *CEN3* (Primer pairs *CEN3R7F* and *CEN3R7R*, SGD co-ordinates 139784 to 140099), *CEN4* (Primer pairs PM80 and PM81, SGD co-ordinates 114795 to 115011), CEN5 (Primer pairs PM84 and PM85, SGD co-ordinates 115323 to 115582).

## Supporting Information

Figure S1 (TIF)

File S1 Supplementary Materials.
Figure S1 Legend. Supplementary Materials And Methods, Strain Constructions, Primer Designations. Supplementary References.
(DOCX)

## References

[1] Onn I, Heidinger-Pauli JM, Guacci V, Unal E, Koshland DE (2008) Sister chromatid cohesion: a simple concept with a complex reality. Annu Rev Cell Dev Biol 24: 105-129. doi:10.1146/annurev.cellbio.24.110707.175350. PubMed: 18616427. 18616427 10.1146/annurev.cellbio.24.110707.175350

[2] Skibbens RV (2008) Mechanisms of sister chromatid pairing. Int Rev Cell Mol Biol 269: 283-339. doi:10.1016/S1937-6448(08)01005-8. PubMed: 18779060. 18779060 10.1016/S1937-6448(08)01005-8

[3] Xiong B, Gerton JL (2010) Regulators of the cohesin network. Annu Rev Biochem 79: 131-153. doi:10.1146/annurev-biochem-061708-092640. PubMed: 20331362. 20331362 10.1146/annurev-biochem-061708-092640

[4] Rudra S, Skibbens RV (2013) Cohesin codes - interpreting chromatin architecture and the many facets of cohesin function. J Cell Sci 126: 31-41. doi:10.1242/jcs.116566. PubMed: 23516328. 23516328 10.1242/jcs.116566 PMC3603509

[5] Krantz ID, McCallum J, DeScipio C, Kaur M, Gillis LA et al. (2004) Cornelia de Lange syndrome is caused by mutations in NIPBL, the human homolog of Drosophila melanogaster Nipped-B. Nat Genet 36: 631-635. doi:10.1038/ng1364. PubMed: 15146186. 15146186 10.1038/ng1364 PMC4902017

[6] Gillis LA, McCallum J, Kaur M, DeScipio C, Yaeger D et al. (2004) NIPBL mutational analysis in 120 individuals with Cornelia de Lange syndrome and evaluation of genotype-phenotype correlations. Am J Hum Genet 75: 610-623. doi:10.1086/424698. PubMed: 15318302. 15318302 10.1086/424698 PMC1182048

[7] Tonkin ET, Wang TJ, Lisgo S, Bamshad MJ, Strachan T (2004) NIPBL, encoding a homolog of fungal Scc2-type sister chromatid cohesion proteins and fly Nipped-B, is mutated in Cornelia de Lange syndrome. Nat Genet 36: 636-641. doi:10.1038/ng1363. PubMed: 15146185. 15146185 10.1038/ng1363

[8] Vega H, Waisfisz Q, Gordillo M, Sakai N, Yanagihara I et al. (2005) Roberts syndrome is caused by mutations in ESCO2, a human homolog of yeast *ECO1* that is essential for the establishment of sister chromatid cohesion. Nat Genet 37: 468-470. doi:10.1038/ng1548. PubMed: 15821733. 15821733 10.1038/ng1548

[9] Schüle B, Oviedo A, Johnston K, Pai S, Francke U (2005) Inactivating mutations in ESCO2 cause SC phocomelia and Roberts syndrome: no phenotype-genotype correlation. Am J Hum Genet 77: 1117-1128. doi:10.1086/498695. PubMed: 16380922. 16380922 10.1086/498695 PMC1285169

[10] Musio A, Selicorni A, Focarelli ML, Gervasini C, Milani D et al. (2006) X-linked Cornelia de Lange syndrome owing to SMC1L1 mutations. Nat Genet 38: 528-530. doi:10.1038/ng1779. PubMed: 16604071. 16604071 10.1038/ng1779

[11] Deardorff MA, Kaur M, Yaeger D, Rampuria A, Korolev S et al. (2007) Mutations in cohesin complex members SMC3 and SMC1A cause a mild variant of Cornelia de Lange syndrome with predominant mental retardation. Am J Hum Genet 80: 485-494. doi:10.1086/511888. PubMed: 17273969. 17273969 10.1086/511888 PMC1821101

[12] Zhang B, Chang J, Fu M, Huang J, Kashyap R et al. (2009) Dosage effects of cohesin regulatory factor PDS5 on mammalian development: implications for cohesinopathies. PLOS ONE 4(5): e5232. doi:10.1371/journal.pone.0005232. PubMed: 19412548. 19412548 10.1371/journal.pone.0005232 PMC2672303

[13] van der Lelij P, Chrzanowska KH, Godthelp BC, Rooimans MA, Oostra AB et al. (2010) Warsaw breakage syndrome, a cohesinopathy associated with mutations in the XPD helicase family member DDX11/ChlR1. Am J Hum Genet 86: 262-266. doi:10.1016/j.ajhg.2010.01.008. PubMed: 20137776. 20137776 10.1016/j.ajhg.2010.01.008 PMC2820174

[14] Levitus M, Waisfisz Q, Godthelp BC, de Vries Y, Hussain S et al. (2005) The DNA helicase BRIP1 is defective in Fanconi anemia complementation group J. Nat Genet 37: 934-935. doi:10.1038/ng1625. PubMed: 16116423. 16116423 10.1038/ng1625

[15] Litman R, Peng M, Jin Z, Zhang F, Zhang J et al. (2005) BACH1 is critical for homologous recombination and appears to be the Fanconi anemia gene product FANCJ. Cancer Cell 8: 255-265. doi:10.1016/j.ccr.2005.08.004. PubMed: 16153896. 16153896 10.1016/j.ccr.2005.08.004

[16] Deardorff MA, Wilde JJ, Albrecht M, Dickinson E, Tennstedt S et al. (2012) RAD21 mutations cause a human cohesinopathy. Am J Hum Genet 90(6): 1014-1027. doi:10.1016/j.ajhg.2012.04.019. PubMed: 22633399. 22633399 10.1016/j.ajhg.2012.04.019 PMC3370273

[17] Deardorff MA, Bando M, Nakato R, Watrin E, Itoh T et al. (2012) HDAC8 mutations in Cornelia de Lange syndrome affect the cohesin acetylation cycle. Nature 489(7415): 313-317. doi:10.1038/nature11316. PubMed: 22885700. 22885700 10.1038/nature11316 PMC3443318

[18] Möckel C, Lammens K, Schele A, Hopfner KP (2012) ATP driven structural changes of the bacterial Mre11:Rad50 catalytic head complex. Nucleic Acids Res 40: 914-927. doi:10.1093/nar/gkr749. PubMed: 21937514. 21937514 10.1093/nar/gkr749 PMC3258140

[19] Schiller CB, Lammens K, Guerini I, Coordes B, Feldmann H et al. (2012) Structure of Mre11-Nbs1 complex yields insights into Ataxia-Telangiectasia-like disease mutations and DNA damage signaling. Nat Struct Mol Biol 19: 693-700. doi:10.1038/nsmb.2323. PubMed: 22705791. 22705791 10.1038/nsmb.2323 PMC3392456

[20] Huang CE, Milutinovich M, Koshland D (2005) Rings, bracelet or snaps: fashionable alternatives for SMC complexes. Philos Trans R Soc Lond B Biol Sci 360: 537-542. doi:10.1098/rstb.2004.1609. PubMed: 15897179. 15897179 10.1098/rstb.2004.1609 PMC1569475

[21] Haering CH, Farcas AM, Arumugam P, Metson J, Nasmyth K (2008) The cohesin ring concatenates sister DNA molecules. Nature 454: 297-301. doi:10.1038/nature07098. PubMed: 18596691. 18596691 10.1038/nature07098

[22] Skibbens RV (2010) Buck the establishment: reinventing sister chromatid cohesion. Trends Cell Biol 20: 507-513. doi:10.1016/j.tcb.2010.06.003. PubMed: 20620062. 20620062 10.1016/j.tcb.2010.06.003

[23] Nasmyth K (2011) Cohesin: a catenase with separate entry and exit gates? Nat Cell Biol 13: 1170-1177. doi:10.1038/ncb2349. PubMed: 21968990. 21968990 10.1038/ncb2349

[24] Ocampo-Hafalla MT, Uhlmann F (2011) Cohesin loading and sliding. J Cell Sci 124: 685-691. doi:10.1242/jcs.073866. PubMed: 21321326. 21321326 10.1242/jcs.073866

[25] Skibbens RV, Corson LB, Koshland D, Hieter P (1999) Ctf7p is essential for sister chromatid cohesion and links mitotic chromosome structure to the DNA replication machinery. Genes Dev 13: 307-319. doi:10.1101/gad.13.3.307. PubMed: 9990855. 9990855 10.1101/gad.13.3.307 PMC316428

[26] Toth A, Ciosk R, Uhlmann F, Galova M, Schleiffer A et al. (1999) Yeast cohesin complex requires a conserved protein, Eco1p(Ctf7), to establish cohesion between sister chromatids during DNA replication. Genes Dev 13: 320-333. 9990856 10.1101/gad.13.3.320 PMC316435

[27] Bellows AM, Kenna MA, Cassimeris L, Skibbens RV (2003) Human EFO1p exhibits acetyltransferase activity and is a unique combination of linker histone and Ctf7p/Eco1p chromatid cohesion establishment domains. Nucleic Acids Res 31: 6334-6343. doi:10.1093/nar/gkg811. PubMed: 14576321. 14576321 10.1093/nar/gkg811 PMC275453

[28] Williams BC, Garrett-Engele CM, Li Z, Williams EV, Rosenman ED et al. (2003) Two putative acetyltransferases, san and deco, are required for establishing sister chromatid cohesion in *Drosophila* . Curr Biol 13: 2025-2036. doi:10.1016/j.cub.2003.11.018. PubMed: 14653991. 14653991 10.1016/j.cub.2003.11.018

[29] Hou F, Zou H (2005) Two human orthologues of Eco1/Ctf7 acetyltransferases are both required for proper sister-chromatid cohesion. Mol Biol Cell 16: 3908-3918. doi:10.1091/mbc.E04-12-1063. PubMed: 15958495. 15958495 10.1091/mbc.E04-12-1063 PMC1182326

[30] Ivanov D, Schleiffer A, Eisenhaber F, Mechtler K, Haering CH et al. (2002) Eco1 is a novel acetyltransferase that can acetylate proteins involved in cohesion. Curr Biol 12: 323-328. doi:10.1016/S0960-9822(02)00681-4. PubMed: 11864574. 11864574 10.1016/s0960-9822(02)00681-4

[31] Rolef Ben-Shahar T, Heeger S, Lehane C, East P, Flynn H et al. (2008) Eco1-dependent cohesin acetylation during establishment of sister chromatid cohesion. Science 321: 563-566. doi:10.1126/science.1157774. PubMed: 18653893. 18653893 10.1126/science.1157774

[32] Unal E, Heidinger-Pauli JM, Kim W, Guacci V, Onn I et al. (2008) A molecular determinant for the establishment of sister chromatid cohesion. Science 321: 566-569. doi:10.1126/science.1157880. PubMed: 18653894. 18653894 10.1126/science.1157880

[33] Zhang J, Shi X, Li Y, Kim BJ, Jia J et al. (2008) Acetylation of Smc3 by Eco1 is required for S phase sister chromatid cohesion in both human and yeast. Mol Cell 31: 143-151. doi:10.1016/j.molcel.2008.06.006. PubMed: 18614053. 18614053 10.1016/j.molcel.2008.06.006

[34] Kenna MA, Skibbens RV (2003) Mechanical link between cohesion establishment and DNA replication: Ctf7p/Eco1p, a cohesion establishment factor, associates with three different Replication Factor C complexes. Mol Cell Biol 23: 2999-3007. doi:10.1128/MCB.23.8.2999-3007.2003. PubMed: 12665596. 12665596 10.1128/MCB.23.8.2999-3007.2003 PMC152568

[35] Moldovan GL, Pfander B, Jentsch S (2006) PCNA controls establishment of sister chromatid cohesion during S phase. Mol Cell 23: 723-732. doi:10.1016/j.molcel.2006.07.007. PubMed: 16934511. 16934511 10.1016/j.molcel.2006.07.007

[36] Milutinovich M, Unal E, Ward C, Skibbens RV, Koshland D (2007) A multi-step pathway for the establishment of sister chromatid cohesion. PLOS Genet 3: e12. doi:10.1371/journal.pgen.0030012. PubMed: 17238288. 17238288 10.1371/journal.pgen.0030012 PMC1779304

[37] Maradeo ME, Skibbens RV (2009) The Elg1-RFC clamp-loading complex performs a role in sister chromatid cohesion. PLOS ONE 4: e4707. doi:10.1371/journal.pone.0004707. PubMed: 19262753. 19262753 10.1371/journal.pone.0004707 PMC2650802

[38] Maradeo ME, Skibbens RV (2010) Replication Factor C complexes play unique pro- and anti-establishment roles in sister chromatid cohesion. PLOS ONE 5: e15381. doi:10.1371/journal.pone.0015381. PubMed: 21060875. 21060875 10.1371/journal.pone.0015381 PMC2965161

[39] Song J, Lafont A, Chen J, Wu FM, Shirahige K et al. (2012) Cohesin acetylation promotes sister chromatid cohesion only in association with the replication machinery. J Biol Chem 287: 34325-34336. doi:10.1074/jbc.M112.400192. PubMed: 22896698. 22896698 10.1074/jbc.M112.400192 PMC3464539

[40] Skibbens RV, Maradeo M, Eastman L (2007) Fork it over: the cohesion establishment factor Ctf7p and DNA replication. J Cell Sci 120: 2471-2477. doi:10.1242/jcs.011999. PubMed: 17646671. 17646671 10.1242/jcs.011999

[41] Uhlmann F, Nasmyth K (1998) Cohesion between sister chromatids must be established during DNA replication. Curr Biol 8: 1095-1101. doi:10.1016/S0960-9822(98)70463-4. PubMed: 9778527. 9778527 10.1016/s0960-9822(98)70463-4

[42] Ciosk R, Shirayama M, Shevchenko A, Tanaka T, Toth A et al. (2000) Cohesin’s binding to chromosomes depends on a separate complex consisting of Scc2 and Scc4 proteins. Mol Cell 5: 243-254. doi:10.1016/S1097-2765(00)80420-7. PubMed: 10882066. 10882066 10.1016/s1097-2765(00)80420-7

[43] Bernard P, Drogat J, Maure JF, Dheur S, Vaur S et al. (2006) A screen for cohesion mutants uncovers Ssl3, the fission yeast counterpart of the cohesin loading factor Scc4. Curr Biol 16: 875-881. doi:10.1016/j.cub.2006.03.037. PubMed: 16682348. 16682348 10.1016/j.cub.2006.03.037

[44] Watrin E, Schleiffer A, Tanaka K, Eisenhaber F, Nasmyth K et al. (2006) Human Scc4 is required for cohesin binding to chromatin, sister-chromatid cohesion, and mitotic progression. Curr Biol 16: 863-874. doi:10.1016/j.cub.2006.03.049. PubMed: 16682347. 16682347 10.1016/j.cub.2006.03.049

[45] Lengronne A, McIntyre J, Katou Y, Kanoh Y, Hopfner KP et al. (2006) Establishment of sister chromatid cohesion at the *S.* *cerevisiae* replication fork. Mol Cell 23: 787-799. doi:10.1016/j.molcel.2006.08.018. PubMed: 16962805. 16962805 10.1016/j.molcel.2006.08.018

[46] Guacci V, Koshland D, Strunnikov A (1997) A direct link between sister chromatid cohesion and chromosome condensation revealed through the analysis of *MCD1* in *S.* *cerevisiae* . Cell 91: 47-57. doi:10.1016/S0092-8674(01)80008-8. PubMed: 9335334. 9335334 10.1016/s0092-8674(01)80008-8 PMC2670185

[47] Michaelis C, Ciosk R, Nasmyth K (1997) Cohesins: chromosomal proteins that prevent premature separation of sister chromatids. Cell 91: 35-45. doi:10.1016/S0092-8674(01)80007-6. PubMed: 9335333. 9335333 10.1016/s0092-8674(01)80007-6

[48] Gruber S, Haering CH, Nasmyth K (2003) Chromosomal cohesin forms a ring. Cell 112: 765-777. doi:10.1016/S0092-8674(03)00162-4. PubMed: 12654244. 12654244 10.1016/s0092-8674(03)00162-4

[49] Haering CH, Löwe J, Hochwagen A, Nasmyth K (2002) Molecular architecture of SMC proteins and the yeast cohesin complex. Mol Cell 9: 773-788. doi:10.1016/S1097-2765(02)00515-4. PubMed: 11983169. 11983169 10.1016/s1097-2765(02)00515-4

[50] Skibbens RV (2004) Chl1p, a DNA helicase-like protein in budding yeast, functions in sister-chromatid cohesion. Genetics 166: 33-42. doi:10.1534/genetics.166.1.33. PubMed: 15020404. 15020404 10.1534/genetics.166.1.33 PMC1470669

[51] Mayer ML, Pot I, Chang M, Xu H, Aneliunas V et al. (2004) Identification of protein complexes required for efficient sister chromatid cohesion. Mol Biol Cell 15: 1736-1745. doi:10.1091/mbc.E03-08-0619. PubMed: 14742714. 14742714 10.1091/mbc.E03-08-0619 PMC379271

[52] Petronczki M, Chwalla B, Siomos MF, Yokobayashi S, Helmhart W et al. (2004) Sister-chromatid cohesion mediated by the alternative RF-CCtf18/Dcc1/Ctf8, the helicase Chl1 and the polymerase-alpha-associated protein Ctf4 is essential for chromatid disjunction during meiosis II. J Cell Sci 117: 3547-3559. doi:10.1242/jcs.01231. PubMed: 15226378. 15226378 10.1242/jcs.01231

[53] Parish JL, Rosa J, Wang X, Lahti JM, Doxsey SJ et al. (2006) The DNA helicase ChlR1 is required for sister chromatid cohesion in mammalian cells. J Cell Sci 119: 4857-4865. doi:10.1242/jcs.03262. PubMed: 17105772. 17105772 10.1242/jcs.03262

[54] Inoue A, Li T, Roby SK, Valentine MB, Inoue M et al. (2007) Loss of ChlR1 helicase in mouse causes lethality due to the accumulation of aneuploid cells generated by cohesion defects and placental malformation. Cell Cycle 6: 1646-1654. doi:10.4161/cc.6.13.4411. PubMed: 17611414. 17611414 10.4161/cc.6.13.4411

[55] Amann J, Kidd VJ, Lahti JM (1997) Characterization of putative human homologues of the yeast chromosome transmission fidelity gene, *CHL1* . J Biol Chem 272: 3823-3832. doi:10.1074/jbc.272.6.3823. PubMed: 9013641. 9013641 10.1074/jbc.272.6.3823

[56] Cantor S, Drapkin R, Zhang F, Lin Y, Han J et al. (2004) The BRCA1-associated protein BACH1 is a DNA helicase targeted by clinically relevant inactivating mutations. Proc Natl Acad Sci U S A 101: 2357-2362. doi:10.1073/pnas.0308717101. PubMed: 14983014. 14983014 10.1073/pnas.0308717101 PMC356955

[57] Cantor SB, Bell DW, Ganesan S, Kass EM, Drapkin R et al. (2001) BACH1, a novel helicase-like protein, interacts directly with BRCA1 and contributes to its DNA repair function. Cell 105: 149-160. doi:10.1016/S0092-8674(01)00304-X. PubMed: 11301010. 11301010 10.1016/s0092-8674(01)00304-x

[58] Peng M, Litman R, Jin Z, Fong G, Cantor SB (2006) BACH1 is a DNA repair protein supporting BRCA1 damage response. Oncogene 25: 2245-2253. doi:10.1038/sj.onc.1209257. PubMed: 16462773. 16462773 10.1038/sj.onc.1209257

[59] Gupta R, Sharma S, Sommers JA, Kenny MK, Cantor SB et al. (2007) FANCJ (BACH1) helicase forms DNA damage inducible foci with Replication Protein A and interacts physically and functionally with the single-stranded DNA-binding protein. Blood 110: 2390-2398. doi:10.1182/blood-2006-11-057273. PubMed: 17596542. 17596542 10.1182/blood-2006-11-057273 PMC1988918

[60] Cantor SB, Andreassen PR (2006) Assessing the link between BACH1 and BRCA1 in the FA pathway. Cell Cycle 5: 164-167. doi:10.4161/cc.5.2.2338. PubMed: 16357529. 16357529 10.4161/cc.5.2.2338

[61] Rafnar T, Gudbjartsson DF, Sulem P, Jonasdottir A, Sigurdsson A et al. (2011) Mutations in BRIP1 confer high risk of ovarian cancer. Nat Genet 43: 1104-1107. doi:10.1038/ng.955. PubMed: 21964575. 21964575 10.1038/ng.955

[62] Farina A, Shin JH, Kim DH, Bermudez VP, Kelman Z et al. (2008) Studies with the human cohesin establishment factor, ChlR1. Association of ChlR1 with Ctf18-RFC and Fen1. J Biol Chem 283: 20925-20936. 18499658 10.1074/jbc.M802696200 PMC2475708

[63] Rudra S, Skibbens RV (2012) Sister chromatid cohesion establishment occurs in concert with lagging strand synthesis. Cell Cycle 11: 2114-2121. doi:10.4161/cc.20547. PubMed: 22592531. 22592531 10.4161/cc.20547 PMC3368863

[64] Ogiwara H, Ui A, Lai MS, Enomoto T, Seki M (2007) Chl1 and Ctf4 are required for damage-induced recombinations. Biochem Biophys Res Commun 354: 222-226. doi:10.1016/j.bbrc.2006.12.185. PubMed: 17222391. 17222391 10.1016/j.bbrc.2006.12.185

[65] Gerring SL, Spencer F, Hieter P (1990) The *CHL1* (*CTF1*) gene product of *Saccharomyces* *cereivisiae* is important for chromosome transmission and normal cell cycle progression in G2/M. EMBO J 9: 4347-4358. PubMed: 2265610. 2265610 10.1002/j.1460-2075.1990.tb07884.x PMC552222

[66] Méndez J, Stillman B (2000) Chromatin association of human origin recognition complex, Cdc6, and minichromosome maintenance proteins during the cell cycle: assembly of prereplication complexes in late mitosis. Mol Cell Biol 20: 8602-8612. doi:10.1128/MCB.20.22.8602-8612.2000. PubMed: 11046155. 11046155 10.1128/mcb.20.22.8602-8612.2000 PMC102165

[67] Leman AR, Noguchi C, Lee CY, Noguchi E (2010) Human Timeless and Tipin stabilize replication forks and facilitate sister-chromatid cohesion. J Cell Sci 123: 660-670. doi:10.1242/jcs.057984. PubMed: 20124417. 20124417 10.1242/jcs.057984 PMC2823575

[68] Warren CD, Eckley DM, Lee MS, Hanna JS, Hughes A et al. (2004) S-phase checkpoint genes safeguard high-fidelity sister chromatid cohesion. Mol Biol Cell 15: 1724-1735. doi:10.1091/mbc.E03-09-0637. PubMed: 14742710. 14742710 10.1091/mbc.E03-09-0637 PMC379270

[69] Gordillo M, Vega H, Trainer AH, Hou F, Sakai N et al. (2008) The molecular mechanism underlying Roberts syndrome involves loss of ESCO2 acetyltransferase activity. Hum Mol Genet 17: 2172-2180. doi:10.1093/hmg/ddn116. PubMed: 18411254. 18411254 10.1093/hmg/ddn116

[70] Laloraya S, Guacci V, Koshland D (2000) Chromosomal addresses of the cohesin component Mcd1p. J Cell Biol 151: 1047-1056. doi:10.1083/jcb.151.5.1047. PubMed: 11086006. 11086006 10.1083/jcb.151.5.1047 PMC2174344

[71] Glynn EF, Megee PC, Yu HG, Mistrot C, Unal E et al. (2004) Genome-wide mapping of the cohesin complex in the yeast *Saccharomyces* *cerevisiae* . PLOS Biol 2: E259. doi:10.1371/journal.pbio.0020259. PubMed: 15309048. 15309048 10.1371/journal.pbio.0020259 PMC490026

[72] Kogut I, Wang J, Guacci V, Mistry RK, Megee PC (2009) The Scc2/Scc4 cohesin loader determines the distribution of cohesin on budding yeast chromosomes. Genes Dev 23: 2345-2357. doi:10.1101/gad.1819409. PubMed: 19797771. 19797771 10.1101/gad.1819409 PMC2758738

[73] Megee PC, Mistrot C, Guacci V, Koshland D (1999) The centromeric sister chromatid cohesion site directs Mcd1p binding to adjacent sequences. Mol Cell 4: 445-450. doi:10.1016/S1097-2765(00)80347-0. PubMed: 10518226. 10518226 10.1016/s1097-2765(00)80347-0

[74] Tanaka T, Cosma MP, Wirth K, Nasmyth K (1999) Identification of cohesin association sites at centromeres and along chromosome arms. Cell 98: 847-858. doi:10.1016/S0092-8674(00)81518-4. PubMed: 10499801. 10499801 10.1016/s0092-8674(00)81518-4

[75] Kitajima TS, Kawashima SA, Watanabe Y (2004) The conserved kinetochore protein shugoshin protects centromeric cohesion during meiosis. Nature 427: 510-517. doi:10.1038/nature02312. PubMed: 14730319. 14730319 10.1038/nature02312

[76] Shintomi K, Hirano T (2009) Releasing cohesin from chromosome arms in early mitosis: opposing actions of Wapl-Pds5 and Sgo1. Genes Dev 23: 2224-2236. doi:10.1101/gad.1844309. PubMed: 19696148. 19696148 10.1101/gad.1844309 PMC2751989

[77] Heidinger-Pauli JM, Mert O, Davenport C, Guacci V, Koshland D (2010) Systematic reduction of cohesin differentially affects chromosome segregation, condensation, and DNA repair. Curr Biol 20: 957-963. doi:10.1016/j.cub.2010.04.018. PubMed: 20451387. 20451387 10.1016/j.cub.2010.04.018 PMC2892909

[78] Vaur S, Feytout A, Vazquez S, Javerzat JP (2012) Pds5 promotes cohesin acetylation and stable cohesin-chromosome interaction. EMBO Rep 13: 645-652. doi:10.1038/embor.2012.72. PubMed: 22640989. 22640989 10.1038/embor.2012.72 PMC3388792

[79] Laha S, Das SP, Hajra S, Sanyal K, Sinha P (2011) Functional characterization of the *Saccharomyces* *cerevisiae* protein Chl1 reveals the role of sister chromatid cohesion in the maintenance of spindle length during S-phase arrest. BMC Genet 12: 83. doi:10.1186/1471-2350-12-83. PubMed: 21943249. 21943249 10.1186/1471-2156-12-83 PMC3190345

[80] Skibbens RV (2000) Holding your own: establishing sister chromatid cohesion. Genome Res 10: 1664-1671. doi:10.1101/gr.153600. PubMed: 11076851. 11076851 10.1101/gr.153600

[81] Lengronne A, Katou Y, Mori S, Yokobayashi S, Kelly GP et al. (2004) Cohesin relocation from sites of chromosomal loading to places of convergent transcription. Nature 430: 573-578. doi:10.1038/nature02742. PubMed: 15229615. 15229615 10.1038/nature02742 PMC2610358

[82] Guacci V, Koshland D (2012) Cohesin-independent segregation of sister chromatids in budding yeast. Mol Biol Cell 23: 729-739. doi:10.1091/mbc.E11-08-0696. PubMed: 22190734. 22190734 10.1091/mbc.E11-08-0696 PMC3279399

[83] Bylund GO, Burgers PM (2005) Replication protein A-directed unloading of PCNA by the Ctf18 cohesion establishment complex. Mol Cell Biol 25: 5445-5455. doi:10.1128/MCB.25.13.5445-5455.2005. PubMed: 15964801. 15964801 10.1128/MCB.25.13.5445-5455.2005 PMC1156988

[84] Mc Intyre J, Muller EG, Weitzer S, Snydsman BE, Davis TN et al. (2007) In vivo analysis of cohesin architecture using FRET in the budding yeast *Saccharomyces* *cerevisiae* . EMBO J 26: 3783-3793. doi:10.1038/sj.emboj.7601793. PubMed: 17660750. 17660750 10.1038/sj.emboj.7601793 PMC1952217

[85] Zheng L, Shen B (2011) Okazaki fragment maturation: nucleases take centre stage. J Mol Cell Biol 3: 23-30. doi:10.1093/jmcb/mjq048. PubMed: 21278448. 21278448 10.1093/jmcb/mjq048 PMC3030970

[86] Gerlich D, Koch B, Dupeux F, Peters JM, Ellenberg J (2006) Live-cell imaging reveals a stable cohesin-chromatin interaction after but not before DNA replication. Curr Biol 16: 1571-1578. doi:10.1016/j.cub.2006.06.068. PubMed: 16890534. 16890534 10.1016/j.cub.2006.06.068

[87] Leman AR, Noguchi E (2013) The Replication Fork: Understanding the eukaryotic replication machinery and the challenges to genome duplication. Genes (Basel) 4: 1-32. doi:10.3390/genes4010001. PubMed: 23599899. 23599899 10.3390/genes4010001 PMC3627427

[88] McNairn AJ, Gerton JL (2009) Intersection of ChIP and FLIP, genomic methods to study the dynamics of the cohesin proteins. Chromosome Res 17: 155-163. doi:10.1007/s10577-008-9007-9. PubMed: 19308698. 19308698 10.1007/s10577-008-9007-9

[89] Gause M, Misulovin Z, Bilyeu A, Dorsett D (2010) Dosage-sensitive regulation of cohesin chromosome binding and dynamics by Nipped-B, Pds5, and Wapl. Mol Cell Biol 30: 4940-4951. doi:10.1128/MCB.00642-10. PubMed: 20696838. 20696838 10.1128/MCB.00642-10 PMC2950535

[90] Onn I, Koshland D (2011) In vitro assembly of physiological cohesin/DNA complexes. Proc Natl Acad Sci U S A 108: 12198-12205. doi:10.1073/pnas.1107504108. PubMed: 21670264. 21670264 10.1073/pnas.1107504108 PMC3145678

[91] Hirota Y, Lahti JM (2000) Characterization of the enzymatic activity of hChlR1, a novel human DNA helicase. Nucleic Acids Res 28: 917-924. doi:10.1093/nar/28.4.917. PubMed: 10648783. 10648783 10.1093/nar/28.4.917 PMC102573

[92] Wu Y, Sommers JA, Khan I, de Winter JP, Brosh RM Jr (2012) Biochemical characterization of Warsaw breakage syndrome helicase. J Biol Chem 287: 1007-1021. doi:10.1074/jbc.M111.276022. PubMed: 22102414. 22102414 10.1074/jbc.M111.276022 PMC3256869

[93] Wu Y, Shin-Ya K, Brosh RM Jr (2008) FANCJ helicase defective in Fanconia anemia and breast cancer unwinds G-quadruplex DNA to defend genomic stability. Mol Cell Biol 28: 4116-4128. doi:10.1128/MCB.02210-07. PubMed: 18426915. 18426915 10.1128/MCB.02210-07 PMC2423121

[94] Wu Y, Brosh RM Jr (2012) DNA helicase and helicase-nuclease enzymes with a conserved iron-sulfur cluster. Nucleic Acids Res 40: 4247-4260. doi:10.1093/nar/gks039. PubMed: 22287629. 22287629 10.1093/nar/gks039 PMC3378879

[95] Maizels N, Gray LT (2013) The G4 genome. PLOS Genet 9: e1003468. 23637633 10.1371/journal.pgen.1003468 PMC3630100

[96] Capo-Chichi JM, Bharti SK, Sommers JA, Yammine T, Chouery E et al. (2013) Identification and biochemical characterization of a novel mutation in DDX11 causing Warsaw breakage syndrome. Hum Mutat 34: 103-107. doi:10.1002/humu.22226. PubMed: 23033317. 23033317 10.1002/humu.22226 PMC4599780

[97] Inoue A, Hyle J, Lechner MS, Lahti JM (2011) Mammalian ChlR1 has a role in heterochromatin organization. Exp Cell Res 317: 2522-2535. doi:10.1016/j.yexcr.2011.08.006. PubMed: 21854770. 21854770 10.1016/j.yexcr.2011.08.006 PMC3184000

[98] Hartman T, Stead K, Koshland D, Guacci V (2000) Pds5p is an essential chromosomal protein required for both sister chromatid cohesion and condensation in *Saccharomyces* *cerevisiae* . J Cell Biol 151: 613-626. doi:10.1083/jcb.151.3.613. PubMed: 11062262. 11062262 10.1083/jcb.151.3.613 PMC2185591

[99] Sutani T, Kawaguchi T, Kanno R, Itoh T, Shirahige K (2009) Budding yeast Wpl1(Rad61)-Pds5 complex counteracts sister chromatid cohesion-establishing reaction. Curr Biol 19: 492-497. doi:10.1016/j.cub.2009.01.062. PubMed: 19268589. 19268589 10.1016/j.cub.2009.01.062

[100] Rowland BD, Roig MB, Nishino T, Kurze A, Uluocak P et al. (2009) Building sister chromatid cohesion: Smc3 acetylation counteracts an antiestablishment activity. Mol Cell 33: 763-774. doi:10.1016/j.molcel.2009.02.028. PubMed: 19328069. 19328069 10.1016/j.molcel.2009.02.028

[101] Morita A, Nakahira K, Hasegawa T, Uchida K, Taniguchi Y et al. (2012) Establishment and characterization of Roberts syndrome and SC phocomelia model medaka (*Oryzias* *latipes*). Dev Growth Differ 54: 588-604. doi:10.1111/j.1440-169X.2012.01362.x. PubMed: 22694322. 22694322 10.1111/j.1440-169X.2012.01362.x

[102] Whelan G, Kreidl E, Peters JM, Eichele G (2012) The non-redundant function of cohesin acetyltransferase Esco2: some answers and new questions. Nucleus 3: 330-334. doi:10.4161/nucl.20440. PubMed: 22614755. 22614755 10.4161/nucl.20440

[103] Gartenberg M (2009) Heterochromatin and the cohesion of sister chromatids. Chromosome Res 17: 229-238. doi:10.1007/s10577-008-9012-z. PubMed: 19308703. 19308703 10.1007/s10577-008-9012-z

[104] Dorsett D (2011) Cohesin: genomic insights into controlling gene transcription and development. Curr Opin Genet Dev 21: 199-206. doi:10.1016/j.gde.2011.01.018. PubMed: 21324671. 21324671 10.1016/j.gde.2011.01.018 PMC3070859

[105] Chen Z, McCrosky S, Guo W, Li H, Gerton JL (2012) A genetic screen to discover pathways affecting cohesin function in *Schizosaccharomyces* *pombe* identifies chromatin effectors. G3 (Bethesda) 2: 1161-1168. 23050226 10.1534/g3.112.003327 PMC3464108

[106] Suhasini AN, Brosh RM Jr (2013) Disease-causing missense mutations in human DNA helicase disorders. Mutat Res 752: 138-152. doi:10.1016/j.mrrev.2012.12.004. PubMed: 23276657. 23276657 10.1016/j.mrrev.2012.12.004 PMC3640642

[107] Borges V, Smith DJ, Whitehouse I, Uhlmann F (2013) An Eco1-independent sister chromatid cohesion establishment pathway in *S.* *cerevisiae* . Chromosoma 122: 121-134. doi:10.1007/s00412-013-0396-y. PubMed: 23334284. 23334284 10.1007/s00412-013-0396-y PMC3608886

[108] Miles J, Formosa T (1992) Evidence that Pob1, a *Saccharomyces* *cerevisiae* protein that binds to DNA polymerase alpha, acts in DNA metabolism in vivo. Mol Cell Biol 12: 5724-5735. PubMed: 1448101. 1448101 10.1128/mcb.12.12.5724-5735.1992 PMC360512

[109] Hanna JS, Kroll ES, Lundblad V, Spencer FA (2001) *Saccharomyces* *cerevisiae* *CTF18* and *CTF4* are required for sister chromatid cohesion. Mol Cell Biol 21: 3144-3158. doi:10.1128/MCB.21.9.3144-3158.2001. PubMed: 11287619. 11287619 10.1128/MCB.21.9.3144-3158.2001 PMC86942

