# Supplementary figures and images for: Chl1 DNA Helicase Regulates Scc2 Deposition Specifically during DNA-Replication in Saccharomyces cerevisiae
Source: PLoS One. 2013 Sep 26;8(9):e75435. doi: 10.1371/journal.pone.0075435 (PMC3784445; doi:10.1371/journal.pone.0075435)

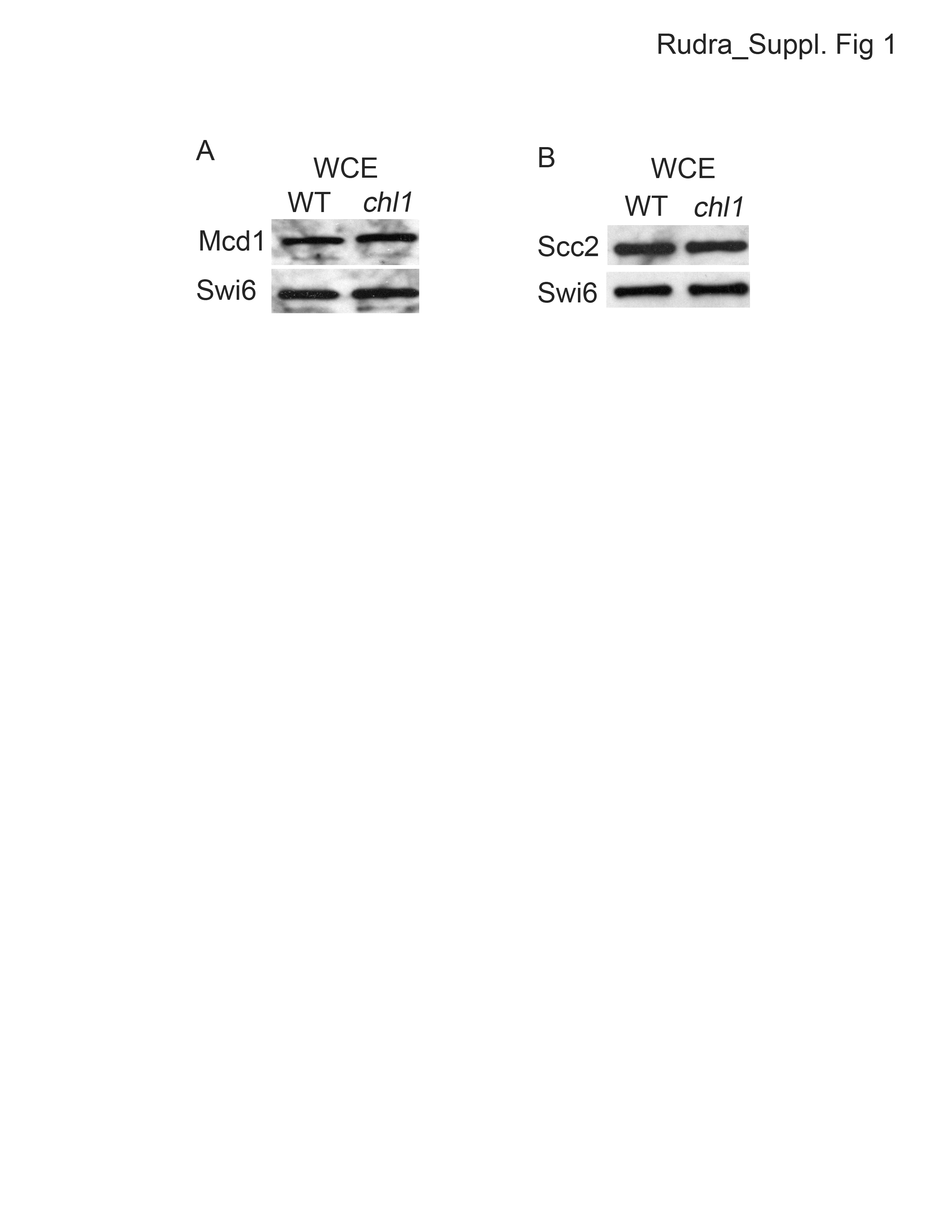

Supplement: Figure S1 — (TIF) [file pone.0075435.s001.tif]
