# Supplementary material for: Chl1 DNA Helicase Regulates Scc2 Deposition Specifically during DNA-Replication in Saccharomyces cerevisiae
Source: PLoS One. 2013 Sep 26;8(9):e75435. doi: 10.1371/journal.pone.0075435 (PMC3784445; doi:10.1371/journal.pone.0075435)
Supplement: File S1 — Supplementary Materials. Figure S1 Legend. Supplementary Materials And Methods, Strain Constructions, Primer Designations. Supplementary References. (DOCX) [file pone.0075435.s002.docx]

**SUPPLEMENTARY MATERIALS**

**Supplemental Figure Legend S1**

Whole cells extracts show identical Mcd1 and Scc2 levels in wild type (YBS 1157, YSR 135) and *chl1* cells (YBS 1175, YSR 138). Wild type and *chl1* cells expressing Mcd1-3HA and Scc2-3HA were lysed and a portion whole cell lysates were solubilized with Laemmli buffer for immunoblotting and the rest processed for chromatin binding. A) Immunoblots of whole cells extracts of wild type and *chl1*cells expressing Mcd1-3HA. b) Immunoblots of whole cell extracts of wild type and *chl1* cells expressing Scc2-3HA.

**Supplemental Materials and Methods**

**Strain constructions**

To construct *fen1*::*KANr* cells, PCR fragments were generated using primers 5’-CGA TGA AAA GCG TTG ACA GCA TAC ATT GGA AAG AAA TAG CGG ATC CCC GGG TTA ATT AA-3’ and 5’- CAA GGT GAA GGA CCA AAA GAA GAA AGT GAA AAA AGA ACC CCC GAA TTC GAG CTC GTT TAA AC-3’ and pFA6a-kanMX6 [1]. The resulting PCR product was transformed into YBS1157. *FEN1::KANr* was confirmed by PCR using primers 5’-GGT GAC TTT CGT TAA TGG GGA-3’ and 5’-GCA AAC GAA TTA CAG CCA GTG-3’.

To construct *SMC3-HA* cells, PCR fragments were generated using primers 5’- GGT TAT TGA GGT CAA TAG AGA AGA AGC AAT CGG ATT CAT TAG AGG TAG CAA TAA ATT CGC TGA ACG GAT CCC CGG GTT AAT TAA -3’ and 5’- TTT AGG TAA GAA GAA GCC AAG TGG TGG ATT TGC ATC ATT AAT AAA AGA TAT TTC AAG AAA AGA ATT CGA GCT CGT TTA AAC -3’ on pFA6a-3HA-*TRP1* [1]. The PR product was transformed into YBS1019. Smc3-HA was confirmed by western blotting and PCR analysis using the primers 5’-GCG GCT CGA GAT TCT TGT TCA ATC GTT GTA ACT CAG C -3’ and 5’- AAC TGC ATG GAG ATG AGT GGT-3’.

To construct *SCC2-3HA* cells, PCR fragments were generated using primers 5’-TCA AAT GGC AAG CTT CTT ACA TAT TTT AGA AAA CAG GTG AAG GAT ACG CGG ATC CCC GGG TTA ATT AA-3’ and 5’-CAG CAT GGA AAA TGC AAA TGC AAA ATG ATT ATT AAT ACT ATG TAT ATT GAA TTC GAG CTC GTT TAA AC-3’ pFA6a-3HA-TRP1 [1] and transformed into YBS1019. Scc2-HA was confirmed by western blotting and PCR analysis using the primers 5’- TCA AAT GGC AAG CTT CTT ACA TAT TTT AGA AAA CAG GTG-3’ and 5’- AAC TGC ATG GAG ATG AGT GGT-3’.

To construct *chl1::HIS3* cells, PCR fragments were generated using the primers 5’- GTA GAA AAC CAG GCT AAA AAC AGT CAC ACT AGT CCA AAA AAC GGA TCC CCG GGT TAA TTA A-3’ and 5’-ATA TAG TAG TAA TCA CAG TAT ACA GGT AAA CGT ATT CCT TGA ATT CGA GCT CGT TTA AAA C-3’ on p-FA5a-His3MX6 [1] and transformed into YSR132 and YSR135. *CHL1::HIS3* was confirmed using primers 5’-TGC CTG GCT GAC TTC TTA GAC-3’ and 5’-CGT GAG CAA ACA ACG GGT AAT-3’. To construct *fen1::HIS3* cells, PCR fragments were generated using primers 5’-CGA TGA AAA GCG TTG ACA GCA TAC ATT GGA AAG AAA TAG CGG ATC CCC GGG TTA ATT AA-3’ and 5’- CAA GGT GAA GGA CCA AAA GAA GAA AGT GAA AAA AGA ACC CCC GAA TTC GAG CTC GTT TAA AC-3’ and p-FA5a-His3MX6 [1]. The resulting PCR product was transformed into YBS132. *FEN1::HIS3* was confirmed by PCR using primers 5’-GGT GAC TTT CGT TAA TGG GGA-3’ and 5’-GCA AAC GAA TTA CAG CCA GTG-3’.

**Primer designations used for CAR sites for centromeres and arm sites along chromosome III**

Arm 1 (Primer pairs DK-EU-25 and DK-EU-26, SGD co-ordinates 194137 to 194479), Arm 2 (Primer pairs DK-EU-29 and DK-EU-29 and DK-EU-30, SGD co-ordinates 195996 to 196386), Arm 3 (Primer pairs DK-EU-33 and DK-EU-34, SGD co-ordinates 198380 to 198762), Arm 4 (Primer pairs *MAT36F* and *MAT36R,* SGD co-ordinates 191257 to 191599), Arm 5 (Primer pairs *MAT37F* and *MAT37R*, SGD co-ordinates 191778 to 192108). *CEN1* (Primer pairs *CEN3L5F* and *CEN3L5R*, SGD co-ordinates 99171 to 99460), *CEN2* (Primer pairs *CEN3L3F* and *CEN3L3R*, SGD co-ordinates 108724 to 109020), *CEN3* (Primer pairs *CEN3R7F* and *CEN3R7R*, SGD co-ordinates 139784 to 140099), *CEN4* (Primer pairs PM80 and PM81, SGD co-ordinates 114795 to 115011), CEN5 (Primer pairs PM84 and PM85, SGD co-ordinates 115323 to 115582).

**SUPPLEMENTARY REFERENCES**

1. Longtine MS, McKenzie A 3rd, Demarini DJ, Shah NG, Wach A, et al. (1998) Additional modules for versatile and economical PCR-based gene deletion and modification in *Saccharomyces cerevisiae*. Yeast 14: 953-961.
